# Cooperation of cancer drivers with regulatory germline variants shapes clinical outcomes

**DOI:** 10.1038/s41467-019-12071-2

**Published:** 2019-09-11

**Authors:** Julian Musa, Florencia Cidre-Aranaz, Marie-Ming Aynaud, Martin F. Orth, Maximilian M. L. Knott, Olivier Mirabeau, Gal Mazor, Mor Varon, Tilman L. B. Hölting, Sandrine Grossetête, Moritz Gartlgruber, Didier Surdez, Julia S. Gerke, Shunya Ohmura, Aruna Marchetto, Marlene Dallmayer, Michaela C. Baldauf, Stefanie Stein, Giuseppina Sannino, Jing Li, Laura Romero-Pérez, Frank Westermann, Wolfgang Hartmann, Uta Dirksen, Melissa Gymrek, Nathaniel D. Anderson, Adam Shlien, Barak Rotblat, Thomas Kirchner, Olivier Delattre, Thomas G. P. Grünewald

**Affiliations:** 1 Max-Eder Research Group for Pediatric Sarcoma Biology, Institute of Pathology, Faculty of Medicine, LMU Munich, Munich, Germany; 2 INSERM U830, Équipe Labellisée LNCC Genetics and Biology of Pediatric Cancers, PSL Research University, SIREDO Oncology Centre, Institut Curie Research Centre, Paris, France; 3 Institute of Pathology, Faculty of Medicine, LMU Munich, Munich, Germany; 4 Department of Life Sciences, Ben-Gurion University of the Negev, Beer-Sheva, Israel; 5 Neuroblastoma Genomics, German Cancer Research Center (DKFZ), Heidelberg, Germany; 6 Division of Translational Pathology, Gerhard-Domagk Institute of Pathology, University Hospital of Münster, Münster, Germany; 7 Department of Pediatric Hematology and Oncology, University Hospital of Essen, Essen, Germany; 8 Department of Medicine, University of California, San Diego, La Jolla, CA USA; 9 Department of Computer Science and Engineering, University of California, San Diego, La Jolla, CA USA; 10 Program in Genetics and Genome Biology, The Hospital for Sick Children, Toronto, ON Canada; 11 Department of Laboratory Medicine and Pathobiology, University of Toronto, Toronto, ON Canada; 12 Department of Paediatric Laboratory Medicine, The Hospital for Sick Children, Toronto, ON Canada; 13 German Cancer Consortium (DKTK), Partner site Munich, Munich, Germany; 14 German Cancer Research Center (DKFZ), Heidelberg, Germany

**Keywords:** Bone cancer, Cancer genetics, Paediatric cancer, Sarcoma

## Abstract

Pediatric malignancies including Ewing sarcoma (EwS) feature a paucity of somatic alterations except for pathognomonic driver-mutations that cannot explain overt variations in clinical outcome. Here, we demonstrate in EwS how cooperation of dominant oncogenes and regulatory germline variants determine tumor growth, patient survival and drug response. Binding of the oncogenic EWSR1-FLI1 fusion transcription factor to a polymorphic enhancer-like DNA element controls expression of the transcription factor MYBL2 mediating these phenotypes. Whole-genome and RNA sequencing reveals that variability at this locus is inherited via the germline and is associated with variable inter-tumoral MYBL2 expression. High MYBL2 levels sensitize EwS cells for inhibition of its upstream activating kinase CDK2 in vitro and in vivo, suggesting MYBL2 as a putative biomarker for anti-CDK2-therapy. Collectively, we establish cooperation of somatic mutations and regulatory germline variants as a major determinant of tumor progression and highlight the importance of integrating the regulatory genome in precision medicine.

## Abstract (web summary)

Interactions between germline variants and somatic mutations is a relatively unexplored topic in cancer. Here, in Ewing sarcoma, the authors show that binding of the oncogenic EWSR1-FLI1 fusion transcription factor to a polymorphic enhancer-like DNA element controls MYBL2, whose high expression correlates with prognosis.

## Introduction

The advent of high-throughput “omics” technologies in oncology enabled assignment of patients to targeted therapies based on somatic mutations in the protein coding genome^1^. However, many childhood cancers including Ewing sarcoma (EwS)—a highly aggressive bone-associated cancer—hardly exhibit any recurrent genetic alteration other than pathognomonic and uniformly expressed driver mutations^2,3^. Yet, these tumors show substantial inter-individual heterogeneity concerning clinical behavior and treatment response, which cannot be solely explained by their few additional (epi-)genetic alterations^3–6^.

Recent studies in humans and model organisms suggested that the effects of a dominant oncogene may depend on variations in the regulatory genome^7–11^. Thus, we hypothesized that oncogenic cooperation of driver-mutations with specific regulatory germline variants may explain inter-individual diversity of clinical outcomes in cancer.

We explore this possibility in EwS, which constitutes a genuine model to study such cooperation for several reasons: First, it is characterized by a simple, nearly diploid genome with a single driver-mutation resulting from chromosomal rearrangements fusing the *EWSR1* gene to various members of the ETS family of transcription factors (in 85% *FLI1*)^2,3,12–14^. Second, EWSR1-FLI1 steers ~40% of its target genes by binding DNA at GGAA-microsatellites, which are thereby converted into potent enhancers^15–18^. Third, the enhancer activity of EWSR1-FLI1-bound GGAA-microsatellites strongly depends on the inter-individually variable number of consecutive GGAA-repeats^16,17,19^. Together, these characteristics provide an ideal framework to analyze how cooperation of a dominant oncogene (here EWSR1-FLI1) with polymorphic germline regulatory elements (here GGAA-microsatellites) influences the expression of disease-promoting genes that could explain clinical diversity in cancer. In this study, we show, in the EwS model, how such cooperation steers the expression of the functionally and clinically relevant EWSR1-FLI1 target gene *MYBL2*, thereby determining tumor growth, patient survival, and drug response.

## Results

### EWSR1-FLI1 regulates MYBL2 via a polymorphic GGAA-microsatellite

**Fig. 1:**
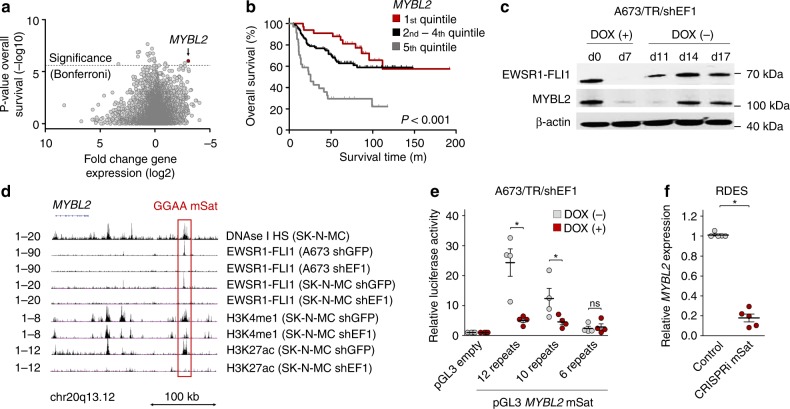
*MYBL2* is a clinically relevant direct EWSR1-FLI1 target gene regulated via a polymorphic GGAA-microsatellite. **a** Integrative analysis of gene expression microarrays of A673/TR/shEF1 cells profiled with/without DOX addition with 166 clinically annotated EwS transcriptomes; *P* values determined via Mantel–Haenszel test. The dashed line indicates the Bonferroni-adjusted *P* value threshold. **b** Kaplan-Meier survival analysis of 166 EwS patients stratified by quintile *MYBL2* expression; *P* value determined via Mantel–Haenszel test. **c** Western blot using antibodies against (EWSR1)-FLI1 and MYBL2 in A673/TR/shEF1 cells. EWSR1-FLI1 was silenced for 7 days by DOX-treatment and re-expressed after DOX-removal for 10 days. Loading control: β-actin. **d** Epigenetic profile of the *MYBL2* locus in indicated EwS cells transduced with either a control shRNA (shGFP) or a specific shRNA against *EWSR1-FLI1* (shEF1) from published DNAse-seq (DNAse I hypersensitivity (HS)) data and ChIP-seq data for EWSR1-FLI1, H3K4me1, and H3K27ac. **e** Reporter assays of *MYBL2*-associated GGAA-microsatellite (mSat) haplotypes in A673/TR/shEF1 cells treated with/without DOX. Horizontal bars represent means, and whiskers represent the SEM, *n* = 4 biologically independent experiments; *P* values determined via one-tailed Mann–Whitney test. **f** Analysis of relative *MYBL2* expression by qRT-PCR in RDES EwS cells with/without CRISPRi-mediated targeting of the *MYBL2*-associated GGAA-microsatellite. Horizontal bars represent means, and whiskers represent the SEM, *n* = 5 biologically independent experiments; *P* values determined via two-tailed Mann–Whitney test. Not significant, ns; *P < 0.05. Source data are provided as a Source Data file

To identify candidate genes with high clinical relevance, we crossed two datasets. The first comprised expression microarrays of A673 EwS cells harboring a doxycycline (DOX)-inducible shRNA against *EWSR1-FLI1* (A673/TR/shEF1) profiled with/without DOX-treatment (Supplementary Data 1). The second comprised 166 transcriptomes of primary EwS with clinical annotation (Supplementary Data 2). We calculated for each gene represented in both datasets the fold change (FC) of its expression after DOX-induced EWSR1-FLI1 knockdown in A673/TR/shEF1 cells and the significance for association with overall survival (OS) stratifying patients by expression quintiles of the corresponding gene. Specifically, the latter analysis was carried out by a custom software (GenEx) that automatically calculates the *P* values for each gene in a given overall survival dataset with matched gene expression data by a Mantel–Haenszel test for patients grouped in the highest versus the lowest expression quintile of the given gene (adjusted for multiple comparisons by the Bonferroni method) (see Methods). This analysis identified *MYBL2* (alias *B-MYB*), encoding a central transcription factor regulating cell proliferation, cell survival, and differentiation^20^, as the top EWSR1-FLI1 upregulated gene, whose high expression was significantly associated with poor OS (nominal *P* = 9.6×10^−7^, Bonferroni-adjusted *P* = 0.018) (Fig. 1a, b; Supplementary Data 3).

The EWSR1-FLI1-dependency of MYBL2 expression was validated in time-course experiments in A673/TR/shEF1 on the mRNA and protein level in vitro and in vivo (Fig. 1c, Supplementary Fig. 1a–c), and in nine additional cultured EwS cell lines (Supplementary Fig. 1d).

Despite this tight regulation of MYBL2 by EWSR1-FLI1, we noted a marked inter-tumor heterogeneity of *MYBL2* mRNA expression in 166 primary EwS (Supplementary Fig. 1e**)** and in an independent cohort of 208 EwS on protein level stained for p-MYBL2 (Supplementary Fig. 1f). Interestingly, *MYBL2* expression did not correlate with minor variations of *EWSR1-FLI1* expression (Supplementary Fig. 1g), suggesting that inter-individual diversity of *MYBL2* transcription may be caused differently.

In accordance, re-analysis of published chromatin immunoprecipitation followed by next-generation sequencing (ChIP-seq) data from A673 and SK-N-MC EwS cells^21,22^ revealed strong signals for EWSR1-FLI1 that mapped to a polymorphic GGAA-microsatellite located ~150 kb telomeric of *MYBL2* (Fig. 1d). In both cell lines, this GGAA-microsatellite exhibited EWSR1-FLI1-dependent epigenetic characteristics of an active enhancer indicated by H3K4me1 and H3K27ac marks (Fig. 1d). The EWSR1-FLI1-dependent enhancer activity of this GGAA-microsatellite was confirmed in reporter assays, for which we cloned fragments of ~880 bp from cell line-derived haplotypes differing in their number of consecutive GGAA-repeats (6, 10, or 12 GGAA-repeats) in the pGL3-Fluc vector. Other regulatory variants in the flanking regions were excluded by whole-genome sequencing (WGS) of the parental cell lines and by Sanger sequencing of the cloned fragments (see Methods). In these assays, we observed a positive correlation of the measured enhancer activity and the number of consecutive GGAA-repeats (Fig. 1e).

To test whether EWSR1-FLI1 prefers haplotypes with more consecutive GGAA-repeats, we carried out ChIP-seq analysis using relatively long reads (single-end 150 bp) for EWSR1-FLI1 in the EwS cell line RDES that is heterozygous at the *MYBL2*-associated GGAA-microsatellite (12 versus 14 consecutive GGAA-repeats). In this analysis, we obtained 31 ChIP-seq reads spanning the entire GGAA-microsatellite. In line with our results from reporter assays (Fig. 1e), 71% of these spanning reads (22/31) mapped to the longer haplotype, whereas only 29% (9/31) mapped to the shorter one (*P* = 0.015).

Applying the haplotype inference and phasing for short tandem repeats (HipSTR)^23^ algorithm on 38 pairs of germline and EwS tumor WGS data covering the *MYBL2*-associated GGAA-microsatellite^6,12^, we identified additional haplotypes with 6–17 consecutive GGAA-repeats (average 13.1 GGAA-repeats). Notably, all haplotypes (76/76) were entirely conserved between germline and tumor DNA (Supplementary Data 4).

We next performed expression quantitative trait locus (eQTL) analysis in 35 primary EwS tumors for which matched gene expression and WGS data were available. Prior reports suggested that more than 13 consecutive GGAA-repeats at EWSR1-FLI1 bound GGAA-microsatellites delineate a critical number beyond which very strong EWSR1-FLI1 binding and enhancer activity can be observed^16,19,24^, which is in agreement with our ChIP-seq analysis showing preferential EWSR1-FLI1 binding to the longer haplotype as stated above. Classifying all haplotypes in either “short” (≤13 GGAA-repeats) or “long” (>13 GGAA-repeats), we detected a significantly higher *MYBL2* expression in EwS tumors with long/long haplotypes compared to those with short/short haplotypes (Supplementary Data 4, Supplementary Fig. 1h).

We further validated the EWSR1-FLI1-mediated regulation of *MYBL2* in time-course EWSR1-FLI1 ChIP-seq and RNA sequencing (RNA-seq) data generated in A673/TR/shEF1 cells^25^. Removal of DOX after suppression of EWSR1-FLI1 for 7 days led to a gradual increase of *MYBL2* transcription that correlated with increasing EWSR1-FLI1 recruitment to this GGAA-microsatellite (*r*_Pearson_ = 0.816). Strikingly, targeting this GGAA-microsatellite by clustered regularly interspaced short palindromic repeats interference (CRISPRi)^26–28^ in highly *MYBL2* expressing RDES cells strongly suppressed *MYBL2* transcription (Fig. 1f) and induced a potentially counter-regulatory upregulation of *EWSR1-FLI1* (Supplementary Fig. 1i). Interestingly, these cells showed a significantly decreased cell growth relative to controls (Supplementary Fig. 1j).

Together, these findings indicate that *MYBL2* is a clinically relevant direct EWSR1-FLI1 target gene, whose expression can be modulated by EWSR1-FLI1 binding to a polymorphic enhancer-like GGAA-microsatellite.

### MYBL2 is critical for proliferation and cell survival of EwS cells

**Fig. 2:**
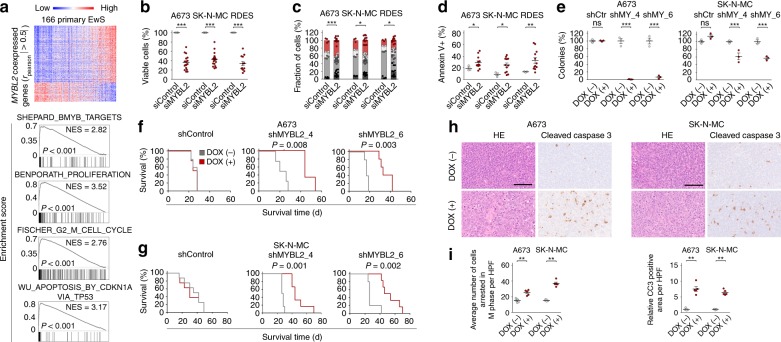
MYBL2 is critical for proliferation and cell survival of EwS cells in vitro and in vivo. **a** Upper: Heat-map of genes whose expression is positively or negatively correlated with *MYBL2* in 166 primary EwS. Lower: GSEA of the same dataset showing selected gene-sets enriched in *MYBL2* high-expressing tumors. **b** Viable cell count 96 h after transfection of three EwS cell lines with either four different specific siRNAs against *MYBL2* (summary of four different siRNAs shown) or a non-targeting siControl. Horizontal bars represent means, and whiskers represent the SEM, *n* ≥ 3 biologically independent experiments. **c** Analysis of cell cycle and cell death (sub G1/G0) 96 h after transfection of three EwS cell lines as described in **b**, using PI staining. Dots show the percentages of cells per experiment delineating cell cycle phases, bars show the fraction of cells (%) in each cell cycle phase, *n* ≥ 3 biologically independent experiments. **d** Analysis of apoptosis 96 h after transfection of three EwS cell lines as described in **b**, using Annexin V/PI staining. Horizontal bars represent means, and whiskers represent the SEM, *n* = 3 biologically independent experiments. **e** Relative colony number of A673 and SK-N-MC cells containing either DOX-inducible specific shRNA constructs directed against MYBL2 (shMY_4 refers to shMYBL2_4 and shMY_6 refers to shMYBL2_6) or a non-targeting shControl (shCtr). Cells were grown either with or without DOX. Horizontal bars represent means, and whiskers the SEM, *n* = 3 biologically independent experiments. **f**, **g** Kaplan–Meier survival analysis of NSG mice xenografted with A673 or SK-N-MC cells with/without DOX-inducible *MYBL2* suppression. Once tumors were palpable, mice were randomized and treated with either vehicle (–) or DOX (+), *n* ≥ 4 animals per condition. *P* values determined via Mantel–Haenszel test. **h** Representative micrographs of xenografts stained with hematoxylin and eosin (HE) or for cleaved caspase 3 (CC3) by IHC. Scale bar is 100 µm. **i** Quantification of cells arrested in M-phase and automated quantification of the picture area positive for CC3 (relative to control) of micrographs described in **h**. Horizontal bars represent means, and whiskers represent the SEM, *n* = 5 samples per condition. ***P < 0.001, **P < 0.01, *P < 0.05; *P* values determined via two-tailed Mann–Whitney test. Source data are provided as a Source Data file

To obtain first clues on the functional role of MYBL2 in primary EwS, we performed gene-set enrichment analysis (GSEA) of *MYBL2* co-expressed genes in 166 EwS tumors. GSEA revealed that *MYBL2* co-expressed genes were strongly enriched in human orthologs of known MYBL2 targets in zebrafish^29^ and in signatures related to proliferation^30^, cell cycle progression^31^, and sensitization to apoptosis mediated by a CDK-inhibiting protein^32^ (Fig. 2a, Supplementary Data 5), suggesting that MYBL2 may constitute a key downstream mediator of EWSR1-FLI1-induced, evolutionary conserved proliferation programs.

To test this hypothesis, we performed *MYBL2* knockdown experiments in A673, SK-N-MC, and RDES EwS cell lines with moderate to high baseline *MYBL2* expression (Supplementary Fig. 2a–c). Using four different siRNAs, we found that *MYBL2* silencing reduced proliferation through blockage of G2/M progression, which was accompanied by increased apoptotic cell death (Fig. 2b–d).

To further explore the function of MYBL2 in EwS growth, we generated DOX-inducible anti-*MYBL2* shRNA expression systems in A673 and SK-N-MC cells using two different shRNAs. In both cell lines, DOX-induced *MYBL2* silencing significantly reduced clonogenic growth in vitro and tumor growth in vivo compared to a non-targeting control shRNA (Fig. 2e–g, Supplementary Fig. 2d–g). In line with our transient knockdown experiments, we observed an increased number of stalled mitoses, indicating G2/M blockage, and more apoptotic tumor cells positive for cleaved caspase 3 in *MYBL2*-silenced xenografts (Fig. 2h, i). Collectively, these findings indicate that MYBL2 is a critical pro-proliferative downstream effector of EWSR1-FLI1 required for proper G2/M transition and cell survival.

### MYBL2 mediates its phenotype via upregulation of *CCNF*, *BIRC5*, and *AURKB*

**Fig. 3:**
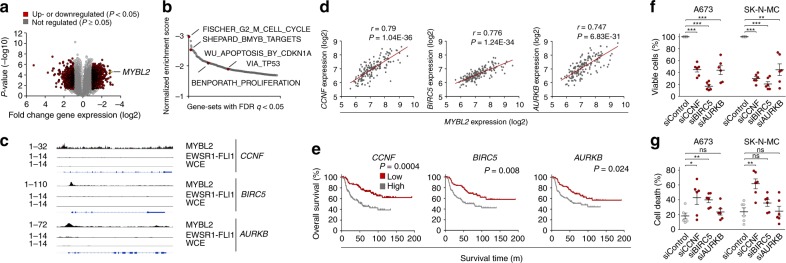
MYBL2 mediates its phenotype via direct upregulation of *CCNF*, *BIRC5*, and *AURKB*. **a** RNA-seq showing differentially expressed genes (DEGs) after siRNA-mediated *MYBL2* knockdown compared to a non-targeting siControl. A summary of three cell lines is shown; *n* = 3 technical replicates per condition. **b** GSEA of RNA-seq data. Displayed are 275 gene-sets downregulated upon *MYBL2* knockdown that had an FDR *q* < 0.05. **c** Analysis of MYBL2 ChIP-seq data from A673 cells showing MYBL2 peaks in the promoters of *CCNF*, *BIRC5*, and *AURKB*. Publicly available EWSR1-FLI1 ChIP-seq data from A673 cells was analyzed to exclude a direct regulation by EWSR1-FLI1. Whole-cell extract (WCE) served as a control. **d** Linear regression of *CCNF*, *BIRC5,* and *AURKB* expression onto *MYBL2* expression in 166 EwS tumors. **e** Kaplan–Meier survival analyses of 166 EwS patients stratified by median expression levels of the indicated gene; *P* values determined via Mantel–Haenszel test. **f** Viable cell count 96 h after transfection of A673 and SK-N-MC cell lines with two different siRNAs directed against either *CCNF*, *BIRC5*, or *AURKB* (summary of two different siRNAs shown) or a non-targeting siControl. Horizontal bars represent means, and whiskers represent the SEM, *n* ≥ 3 biologically independent experiments. **g** Measurement of cell death using Trypan blue positivity 96 h after transfection of A673 and SK-N-MC cells transfected as described in **f**. Horizontal bars represent means, and whiskers represent the SEM, *n* ≥ 3 biologically independent experiments. ***P < 0.001, **P < 0.01, *P < 0.05; *P* values determined via two-tailed Mann–Whitney test. Source data are provided as a Source Data file

To identify potential direct MYBL2 targets that could explain its pro-proliferative effect, we sequenced RNA of three EwS cell lines with/without *MYBL2* knockdown (Fig. 3a). Consistent with our enrichment analyses in primary EwS and functional experiments, GSEA of the identified differentially expressed genes (DEGs) showed that *MYBL2* suppression leads to a strong downregulation of the same gene sets comprising human orthologs of zebrafish MYBL2 targets and identical proliferation, cell cycle, and sensitization to CDK-inhibitor mediated apoptosis gene signatures (Fig. 3b, Supplementary Fig. 3a, Supplementary Data 6).

We then focused on the 76 most significantly DEGs (mean log2 FC |≥1.5|, Bonferroni-adjusted *P* < 0.05), of which representative genes were validated by quantitative real time PCR (qRT-PCR) (Supplementary Fig. 3b, c, Supplementary Data 7). ChIP-seq analysis using a specific anti-MYBL2 antibody revealed that 50 of these 76 DEGs (66%) showed evidence for MYBL2 promoter-binding (Fig. 3c, Supplementary Data 8). Using microarray data of 166 patient tumors in which 92% of these direct MYBL2 targets were represented enabled correlation of their expression levels with that of *MYBL2* and with OS of patients stratified by median expression of the corresponding gene (Supplementary Data 9, 10). Among these genes, *CCNF*, *BIRC5*, and *AURKB* stood out for being highly significantly co-expressed with *MYBL2* (Bonferroni-adjusted *P* < 0.05, *r*_Pearson_ ≥ 0.7) (Fig. 3d), and associated with poor OS (Fig. 3e). To investigate their functional role, we individually knocked down either gene using two specific siRNAs in two different EwS cell lines (Supplementary Fig. 3d) and assessed proliferation and cell viability in vitro. Strikingly, knockdown of these genes broadly phenocopied the anti-proliferative and anti-survival effect of *MYBL2* silencing (Fig. 3f, g), suggesting that they may constitute important mediators of the pro-proliferative EWSR1-FLI1/MYBL2 transcriptional program. However, as other functionally relevant genes (e.g. *MKI67, KIF20A, PIF1*) are also regulated by MYBL2 (Supplementary Fig. 3c), it is conceivable that other genes may contribute to the phenotype of MYBL2.

### High MYBL2 levels sensitize EwS cells toward CDK2 inhibition

**Fig. 4:**
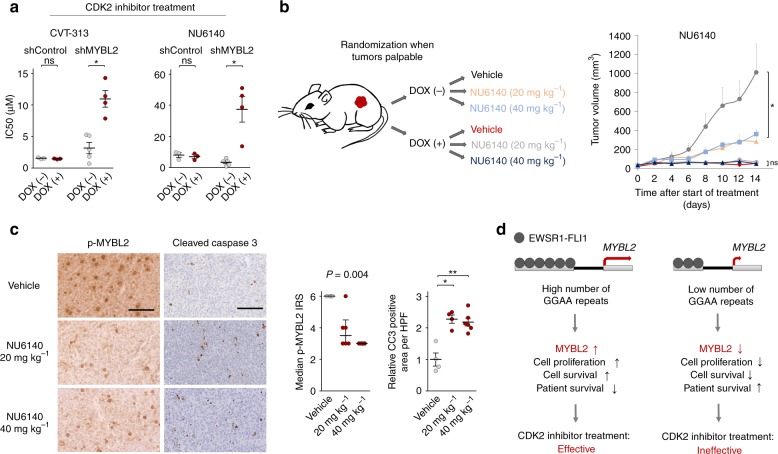
High MYBL2 expression levels sensitize EwS cells toward treatment with CDK2 inhibitors in vitro and in vivo. **a** Analysis of IC50 of CDK2 inhibitors CVT-313 and NU6140 in A673 cells containing either DOX-inducible specific shMYBL2 or non-targeting shControl constructs. Horizontal bars represent means, and whiskers represent the SEM, *n* ≥ 3 biologically independent experiments; *P* values determined via two-tailed Mann–Whitney test. **b** Left: Schematic of the experimental setting of CDK2 inhibitor treatment (NU6140) in vivo. NSG mice were xenografted with A673 cells containing a DOX-inducible shMYBL2 construct, treated with/without DOX and either vehicle or NU6140 in a dose of 20 mg kg^−1^ or 40 mg kg^−1^. Mice were randomized to the treatment groups when tumors were palpable. Right: For each condition the mean tumor volume and SEM of 4–6 mice over the time of treatment are shown; *P* values determined via two-tailed Mann–Whitney test. **c** Left: Representative IHC micrographs of p-MYBL2 and cleaved caspase 3 (CC3) staining of A673/TR/shMYBL2 xenografts (DOX (–)) treated with either vehicle or NU6140. Scale bar is 100 µm. Right: Quantification of positivity for p-MYBL2 and CC3, respectively. Horizontal bars represent medians or means, and whiskers interquartile ranges or SEM for p-MYBL2 or CC3, respectively, *n*≥4 samples per condition; *P* values determined via Kruskal–Wallis test (p-MYBL2) or two-tailed Mann–Whitney test (CC3). **d** Model of EWSR1-FLI1-dependent regulation of *MYBL2* via the *MYBL2*-associated GGAA-microsatellite in EwS. Not significant, ns; ***P < 0.001, **P < 0.01, *P < 0.05. Source data are provided as a Source Data file

As there are—to the best of our knowledge—currently no direct MYBL2 inhibitors available, we reasoned that targeting its major upstream cyclin dependent kinase, CDK2, which activates MYBL2 through phosphorylation^20^, may offer a new therapeutic option for EwS patients with high MYBL2 expression. To test this possibility, we treated EwS cells with two small-molecule CDK2 inhibitors (CVT-313 and NU6140). While both inhibitors strongly reduced growth of A673 EwS cells at the lower micro-molar range, sensitivity toward them was dramatically diminished when *MYBL2* was suppressed (Fig. 4a). Such differential effect was not observed in control cells expressing a non-targeting shRNA (Fig. 4a). Notably, NU6140 is a dual inhibitor of CDK2 and the major downstream MYBL2 target AURKB. Since this inhibitor enabled to specifically target EwS cells up- and downstream of MYBL2, we tested its effect on EwS growth in vivo. Treatment of NOD/scid/gamma (NSG) mice with NU6140 significantly (*P* < 0.05) reduced growth of EwS xenografts compared to vehicle (DMSO) (Fig. 4b), and was accompanied by reduced levels of phosphorylated MYBL2 and increased apoptotic cell death (Fig. 4c). However, this inhibitor had no additional effect on growth of xenografts with silenced *MYBL2* expression (Fig. 4b), suggesting that MYBL2 is important for the anti-proliferative effect of CDK2 inhibitors. Consistently, different EwS cell lines with high *MYBL2* levels showed higher sensitivity toward NU6140 than a EwS cell line with constitutively low *MYBL2* expression (Supplementary Fig. 4a, b). A similar effect on growth of A673 EwS xenografts was observed using the CDK2 inhibitor CVT-313 (Supplementary Fig. 4c, e). Since we neither observed significant weight loss (Supplementary Fig. 4d) nor histomorphological changes in inner organs in mice treated for 14 days with up to 40 mg kg^−1^ of either inhibitor, these results indicated that CDK2 inhibition can safely impair growth of EwS tumors and that MYBL2 may serve as a biomarker to predict its efficacy.

Interestingly, we observed in A673/TR/shEF1 cells that *CDK2* appears to be moderately upregulated by EWSR1-FLI1 (Supplementary Fig. 4f), and found evidence for binding of EWSR1-FLI1 at the *CDK2* locus in EwS cells (Supplementary Fig. 4g). However, whether EWSR1-FLI1 regulates *CDK2* expression directly or indirectly remains to be elucidated in future studies.

## Discussion

Collectively, our discoveries made in an aggressive childhood cancer exemplify how oncogenic cooperation between a cancer driver-mutation (here EWSR1-FLI1) and a regulatory germline variant (here a polymorphic enhancer-like GGAA microsatellite) can create a major source of inter-tumor heterogeneity determining clinical outcome and drug response through modulation of a druggable key downstream player (Fig. 4d).

To explore the possibility of such oncogenic cooperation in EwS beyond *MYBL2*, we analyzed the top five additional hits of our initial screen whose high expression was associated with worse patient overall survival (*EXO1*, *C1ORF112*, *ESPL1*, *HJURP*, *RAD54L*; Supplementary Data 3) for the presence of EWSR1-FLI1 bound GGAA-microsatellites or ETS-like binding motifs in the vicinity of these genes, and in that case also possible eQTL effects. While no EWSR1-FLI1 binding was observed at the *ESPL1* locus, we found evidence for EWSR1-FLI1 binding at GGAA-microsatellites or ETS-like single GGAA-motifs at the other loci. However, most of these EWSR1-FLI1-binding sites did not show genetic variability in WGS data from primary EwS samples, and if so, they appeared to have no eQTL properties (Supplementary Fig. 5), which may further support the special role of *MYBL2* in EwS.

Our results suggest that cooperation between disease-promoting somatic mutations and regulatory germline variants could constitute a general mechanism to explain diversity of disease phenotypes, possibly beyond cancer. In line with this idea, recent reports for neurodegenerative and metabolic diseases showed that the same disease-causing somatic event/mutation can induce distinct phenotypes depending on (inherited) variations in regulatory elements^7,33,34^. We anticipate that our findings made in the EwS model are translatable to other malignancies, and propose that integration of the regulatory genome in the process of developing new predictive markers and therapeutic strategies is necessary to refine and fully exploit “omics”-based precision medicine.

## Methods

### Provenience of cell lines and cell culture conditions

A673 and HEK293T cells were purchased from American Type Culture Collection (ATCC). MHH-ES1, RDES, RH1, SK-ES1, and SK-N-MC cells were provided by the German Collection of Microorganisms and Cell  Cultures (DSMZ). TC-32, TC-71, and CHLA-10 cells were kindly provided by the Children’s Oncology Group (COG) and EW1, EW3, EW7, EW16, EW17, EW18, EW22, EW23, EW24, LAP35, MIC, ORS, POE, STA-ET1, STA-ET8 cells were provided by O. Delattre (Institute Curie, Paris). A673/TR/shEF1 cells were kindly provided by J. Alonso (Madrid, Spain)^35^. The SK-N-MC cell line is listed in the database of commonly misidentified cell lines, ICLAC (http://iclac.org/databases/cross-contaminations), as it was initially described to be a neuroblastoma cell line. Indeed, it is a EwS cell line expressing the pathognomonic fusion oncogene *EWSR1-FLI1*. All cell lines were grown in humidified atmosphere at 37 °C and 5% CO_2_. Cells were cultured in RPMI 1640 medium supplemented with stable glutamine (Biochrom), 10% tetracycline-free FCS (Sigma-Aldrich), 100 Uml^−1^ penicillin (Biochrom), and 100 µg ml^−1^ streptomycin (Biochrom). Cells were routinely checked by nested PCR for mycoplasma infection, and their purity was confirmed by STR-profiling and, if applicable, by PCR-based detection of specific fusion oncogenes.

### DNA/RNA extraction, reverse transcription, and qRT-PCR

DNA was extracted with the NucleoSpin Tissue kit (Macherey-Nagel); plasmid DNA was extracted from bacteria with the PureYield kit (Promega). RNA extraction was performed with the NucleoSpin RNA kit (Macherey-Nagel) and RNA was reverse transcribed using the High-Capacity cDNA Reverse Transcription kit (Applied Biosystems). qRT-PCRs were performed using SYBR Select Master Mix (Applied Biosystems) and reactions were run on a Bio-Rad CFX Connect instrument and analyzed using the Bio-Rad CFX Manager 3.1 software. All oligonucleotides were purchased from MWG Eurofins Genomics. For primer sequences see Supplementary Data 11.

### Transient transfection

For siRNA transfection, cells were seeded in a six-well plate at a density of 1.5 × 10^5^ per well in 1.6 ml of growth medium. The cells were transfected with either a negative control non-targeting siRNA (Sigma-Aldrich MISSION siRNA Universal Negative Control #1) or specific siRNAs (25–65 nM, depending on the cell line and the siRNA) and HiPerfect (Qiagen). Cells were retransfected 48 h after the first transfection and harvested 96 h after the first transfection. siRNA sequences are given in Supplementary Data 11. For plasmid transfection, cells were seeded in a six-well plate at a density of 2 × 10^5^ per well in 1.8 ml of growth medium. Plasmids were transfected with Lipofectamine LTX and Plus Reagent (Invitrogen). The pGL3 vector used for reporter assays has been described before^16^.

### Doxycycline (DOX)-inducible shRNA constructs

Either a non-targeting negative control shRNA (MWG Eurofins Genomics) or specific shRNAs targeting *EWSR1-FLI1* or *MYBL2* (both MWG Eurofins Genomics) were cloned in the pLKO-Tet-on-all-in-one system^36^. Oligonucleotide sequences are given in Supplementary Data 11. Lentiviruses were produced in HEK293T cells. A673 and SK-N-MC EwS cells were infected with respective lentiviruses and selected with 1.5  µg ml^−1^ puromycin (Invivogen). After single-cell cloning, knockdown efficacy of individual clones was assessed by qRT-PCR 48 h after addition of DOX (1 µg ml^−1^; Sigma-Aldrich).

### DNA constructs and reporter assays

MYBL2-associated GGAA-microsatellites (with ~440 bp 5′ and 3′ flanking regions) from three EwS cell lines were PCR-cloned upstream of the SV40 minimal promoter into the pGL3-Fluc vector (Promega)^16^. Primer sequences are given in Supplementary Data 11. The presence of additional variants devoid of the GGAA-microsatellite was ruled out by WGS of the parental cell lines and Sanger sequencing of the cloned fragments. A673/TR/shEF1 cells (2 × 10^5^ per well) were transfected with the *Firefly* pGL3-Fluc vector containing respective microsatellites and the *Renilla* pGL3-Rluc vector (Promega) (ratio 100:1) in a six-well plate with 1.8 ml of growth medium. Four hours after transfection, transfection medium was replaced by medium with/without DOX (1 µg ml^−1^; Sigma-Aldrich). Cells were lysed and assayed with a dual luciferase assay system (Berthold) after 72 h. *Firefly* luciferase activity was normalized to that of *Renilla*.

### CRISPR interference (CRISPRi) and analysis of cell growth

Due to the lack of functional DNAse, CRISPRi does not cause a knockout of the targeted DNA sequence, but blocks protein binding to it^26,28^. For the reported experiments, a DNAse-dead CAS9 (dCAS9) fused to the KRAB effector domain, which promotes an inhibiting chromatin state, is targeted to the genomic region of interest by specific gRNAs to silence the activity of a given enhancer^26,28^. To achieve this, we used a pHAGE TRE dCas9-KRAB vector (Addgene #50917) and a pLKO.1-puro U6 sgRNA BfuAI large stuffer vector (Addgene #52628), the latter containing either two gRNAs, targeting sequences adjacent to the *MYBL2*-associated GGAA-microsatellite, or a scrambled control (Supplementary Data 11). Lentivirus production was performed in HEK293T cells. RDES EwS cells were infected with the respective lentiviruses and selected with 1 µg ml^−1^ puromycin and 1.5 µg ml^−1^ G418 (both Invivogen). The cells were induced with DOX (1 μg ml^−1^; Sigma-Aldrich) for 5 days, after which *MYBL2* and *EWSR1-FLI1* levels were measured by qRT-PCR.

For measurement of cell growth, cells were grown in medium containing selection antibiotics and DOX (2 µg ml^−1^) for 14 days as described^37^. Thereafter, 8 × 10^4^ cells/well were plated in quadruplicate wells of 24-well plates in the presence of DOX. After four additional days, cells were washed and fixed with trichloroacetic acid for 1 h at 4 °C. Then, plates were washed with phosphate-buffered saline (PBS), air dried, and cells were stained with crystal violet (Sigma-Aldrich) for 30 min. Surplus crystal violet was removed by rinsing the plates with PBS. Cell-bound crystal violet was dissolved in 10% acetic acid, and optical density was measured at 595 nm in a DS-11 spectrophotometer (DeNovix Inc.).

### Western blot

Protein from A673/TR/shEF1 cells was extracted at d0, d7, d11, d14, and d17 with RIPA and anti-protease cocktail (Roche). Western blots were performed following routine protocols and specific band detection was achieved by the use of rabbit monoclonal anti-FLI1 antibody (1:1000, ab133485; Abcam)^38^, rabbit polyclonal anti-MYBL2 antibody (1:500, sc-725; Santa Cruz)^39^, and mouse monoclonal anti-ß-actin (1:10,000, A-5316; Sigma-Aldrich). Anti-rabbit IgG horseradish peroxidase-coupled antibody (1:3000, Amersham Bioscience) and anti-mouse IgG horseradish peroxidase coupled antibody (1:3,000; Amersham Bioscience) was used as secondary antibody. Proteins were visualized using chemiluminescence (Pierce ECL Western blot chemiluminescent substrate; Thermo Fisher Scientific).

### Proliferation assays

Cells were seeded in a six-well plate at a density of 1.5 × 10^5^ per well in 1.6 ml of growth medium. The cells were transfected with either a negative control non-targeting siRNA in duplicate wells (Sigma-Aldrich MISSION siRNA Universal Negative Control #1) or up to four specific siRNAs (MWG Eurofins Genomics) (25–65 nM, depending on the cell line and siRNA) using HiPerfect (Qiagen). siRNA sequences are given in Supplementary Data 11. Retransfection was performed 48 h after the first transfection. Ninety-six hours after the first transfection, cells were harvested (including supernatant), stained with Trypan blue (Sigma-Aldrich), and counted in a standardized hemocytometer (C-Chip, NanoEnTek).

### Analysis of cell cycle and apoptosis

Analysis of cell cycle phases was performed by propidium iodide (PI) (Sigma-Aldrich) staining. Cells were transfected with siRNAs equivalently to the proliferation assays (see above), harvested after 96 h (including supernatant), fixed in ethanol (70%) at 4 °C, and stained with PI solution (50 µg ml^−1^, with 20 µg ml^−1^ RNAse A (Invitrogen)). Analysis of apoptosis has been performed by combined Annexin V-FITC/PI staining (BD Pharmingen FITC Annexin V Apoptosis Detection Kit II; BD Biosciences). Cells were transfected with siRNAs equivalently to the proliferation assays (see above) and harvested after 96 h (including supernatant). The samples were assayed on an Accuri C6 flow cytometer and analyzed with the Accuri C6 CFlow Plus software. An example of the gating strategy is given in Supplementary Fig. 6.

### Colony-forming assays

A673 and SK-N-MC cells containing either a DOX-inducible non-targeting control shRNA or *MYBL2*-targeting specific shRNAs were seeded in triplicate wells of a 12-well plate at a density of 500 cells (A673) or 1000 cells (SK-N-MC) per well in 2 ml of growth medium. Cells were grown with/without DOX (1 µg ml^−1^; Sigma-Aldrich) for 10–14 days depending on the cell line and afterwards stained with crystal violet (Sigma-Aldrich). Colony number was determined on scanned plates using Fiji (ImageJ)^40,41^.

### cDNA library and RNA sequencing (RNA-seq)

A673, SK-N-MC, and RDES EwS cell lines were transfected in triplicates with either a negative control non-targeting siRNA or a specific siRNA targeting *MYBL2* (siMYBL2_1). Total RNA was extracted using the NucleoSpin II kit (Macherey-Nagel). Complementary DNA libraries were sequenced with an Illumina HiSeq2500 instrument using 150 bp paired-end sequencing. Obtained reads were aligned on the human genome (hg19) using TopHat (version 2.0.6)^42^. Counting of reads on annotated genes from the GRCh37 gene build was done using htseq-count (v. HTSeq-0.5.3p9)^43^ with the following parameters: htseq-count -a 10 -q -s no -m union. Sample-to-sample normalization and differential expression analyses were performed using the R package DESeq2 (v.1.18.0)^44^. RNA-seq data were deposited at the Gene Expression Omnibus (GEO; accession code GSE119972).

### Chromatin immunoprecipitation and sequencing (ChIP-seq)

DNA–protein cross-linking was done in the presence of 1% of paraformaldehyde on 12 × 10^6^ A673 or 4 × 10^6^ RDES cells, respectively, for each condition for 10 min. Cell lysis, chromatin shearing, immunoprecipitation, and DNA purification were performed with reagents from iDeal ChIP-seq kit for Transcription Factors (Diagenode, ref: C01010054). Chromatin shearing was carried out in a Bioruptor (Diagenode) using 20 cycles of sonication (30 s high, 30 s off) in TPX tubes (Diagenode, ref: 50001). For immunoprecipitation of activated MYBL2, 2 µg of a monoclonal ChIP-grade rabbit anti-p-MYBL2 antibody (Abcam, ab76009, lot GR113270–6)^45^ were used. MYBL2 ChIP and input were sequenced on an Illumina HiSeq2500 instrument (100 bp single-end). For immunoprecipitation of EWSR1-FLI1, 2 µg of a polyclonal ChIP-grade rabbit anti-FLI1 antibody (Abcam, ab15289, lot GR293950-1)^18^ was used. EWSR1-FLI1 ChIP and input were sequenced on an Illumina HiSeq2500 instrument (150 bp single-end). ChIP-seq reads were aligned to the human genome (hg19 version) with Bowtie2 (ref. ^46^). Peaks were called with MACS2 with option narrow^47^. To normalize, we took the input dataset from the same cell line. PAVIS was used for peak annotation and visualization^48^. For analysis of the number of EWSR1-FLI1 ChIP-seq reads spanning the different haplotypes of the *MYBL2*-associated GGAA-microsatellite in RDES cells, exclusively the spanning reads were extracted from the BAM file with SAMtools, and mapped to the corresponding haplotype according to CIGAR scores^49^. Significance levels were calculated using a binomial test (*p* = 0.5). ChIP-seq data concerning MYBL2 were deposited at the GEO (accession code GSE119972).

### Analysis of published ChIP-seq and DNAse-seq data

Publicly available ENCODE SK-N-MC DNAse-seq data (GSM736570) and pre-processed A673 and SK-N-MC ChIP-seq data (GSE61944) were retrieved from the GEO and displayed in the UCSC genome browser. Samples used: GSM1517544 SK-N-MC_shGFP_48h_FLI1; GSM1517553 SK-N-MC_shFLI1_48h_FLI1; GSM1517569 A673_shGFP_48h_FLI1; GSM1517572 A673_shFLI1_48h_FLI1; GSM1517548 SK-N-MC_shGFP_96h_H3K4me1; GSM1517557 SK-N-MC_shFLI1_96h_H3K4me1; GSM1517545 SK-N-MC_shGFP_48h_H3K27ac; GSM1517554 SK-N-MC_shFLI1_48h_H3K27ac; GSM1517568 A673 whole-cell extract (WCE).

### CDK2 inhibitor assays in vitro

Cells were seeded in a 96-well plate at a density of 5 × 10^3^ per well. In case of cells containing DOX-inducible constructs, cells were treated with/without DOX (1 µg ml^−1^; Sigma-Aldrich). Twenty-four hours after seeding or pre-incubation with DOX, respectively, CDK2 inhibitors (CVT-313 or NU6140; Merck and Tocris) were added in serially diluted concentrations ranging from 0.001 to 100 µM. Each well contained an equal concentration of 0.5% DMSO (Sigma-Aldrich). Cells only treated with 0.5% of DMSO served as a control. After 72 h of inhibitor treatment, the plates were assayed on a Thermo Fisher Varioskan plate reader after incubation with Resazurin (20 µg ml^−1^; Sigma-Aldrich) for 6 h.

### Xenotransplantation and CDK2 inhibitor treatment in vivo

All mouse experiments were approved by the local authorities in compliance with all relevant ethical regulations (including, but not limited to, tumor size). Sample size was predetermined using power calculations with *β* = 0.8 and *α* = 0.05 based on preliminary data and in compliance with the 3 R system (replacement, reduction, refinement). 3 × 10^6^ A673 and SK-N-MC cells, containing either a DOX-inducible negative control shRNA or specific shRNAs against *EWSR1-FLI1* or *MYBL2*, were injected subcutaneously with a 1:1 mix of PBS (Biochrom) and Geltrex (LDEV-Free Reduced Growth Factor Basement Membrane Matrix, Thermo Fisher Scientific; max volume 100 µl) in the right flanks of 3–9 months old female or male NSG mice (Charles River Laboratories). For shRNA sequences see Supplementary Data 11. When tumors were palpable, mice were randomized to the control group (17.5 mg ml^−1^ sucrose (Sigma-Aldrich) in drinking water) or the treatment group (2 mg ml^−1^ DOX (Beladox, bela-pharm) and 50 mg ml^−1^ sucrose (Sigma-Aldrich) in drinking water). Tumor size was measured with a caliper every 2 days and tumor volume was calculated as *V* *=* *a* *×* *b*^2^/2 with *a* being the largest diameter and *b* being the smallest diameter. Once the tumors reached a volume of 1500 mm^3^ respective mice were sacrificed by cervical dislocation. Other humane endpoints were determined as follows: Ulcerated tumors, loss of 20% body weight, constant curved or crouched body posture, bloody diarrhea or rectal prolapse, abnormal breathing, severe dehydration, visible abdominal distention, obese Body Condition Scores (BCS), apathy, and self-isolation. For CDK2 inhibitor treatment in vivo, cells were injected as described above. When tumors were palpable, mice were assigned to either the vehicle (DSMO) or a treatment group (20 or 40 mg kg^−1^), each with or without addition of DOX to the drinking water (2 mg ml^−1^ DOX; Beladox, bela-pharm). In case of CVT-313 (Tocris) treatment, DOX was not applied. The CDK2 inhibitors NU6140 or CVT-313 (both Tocris) were administered i.p. for 12 days, with a break of 1 day every 4 days of treatment. The experimental endpoint was predetermined as 14 days after first injection of either inhibitor, or if humane endpoints as described above were reached before. To check histomorphological changes of inner organs upon CDK2 inhibitor treatment, we examined hematoxylin and eosin (HE) stained slides of heart, lungs, liver, stomach, pancreas, intestines, kidneys, adrenal glands, bone marrow, and spleen from treated and non-treated mice. Tumor tissues were subjected to HE staining, as well as immunohistochemical staining for p-MYBL2 and cleaved caspase 3 (as described below). Animal experiments were approved by the government of Upper Bavaria and conducted in accordance with ARRIVE guidelines, recommendations of the European Community (86/609/EEC), and UKCCCR (guidelines for the welfare and use of animals in cancer research).

### Survival analysis

Microarray data of 166 primary EwS tumors (GSE63157, GSE34620, GSE12102, GSE17618) for which clinical annotations were available were downloaded from the GEO. Data were either generated on Affymetrix HG-U133Plus2.0 or on Affymetrix HuEx-1.0-st microarray chips and separately normalized by RMA using custom brainarray chip description files (CDF, v20). ComBat was used to remove batch effects^50,51^. Patients were stratified by their quintile or median intra-tumoral gene expression levels. Mantel–Haenszel test was performed to calculate significance levels, using either a custom code (GenEx) for batch queries or GraphPad PRISM version 5 for individual genes (GraphPad Software Inc., CA, USA). *P* values < 0.05 were considered as statistically significant. Survival data were crossed with gene expression microarray data (Affymetrix HG-U133A2.0) generated in A673/TR/shEF1 cells (GSE27524; 53 h DOX-treatment), which were normalized as described above (RMA with brainarray CDF, v19).

### Gene-set enrichment analysis

Using the Affymetrix gene expression dataset comprising 166 primary EwS patients, enrichment of gene-sets that are among *MYBL2* co-regulated genes were identified by ranking of Pearson´s correlation coefficient of the expression of every gene with *MYBL2* expression and performance of a pre-ranked GSEA with 1000 permutations^52^. Using the RNA-seq dataset containing DEGs after siRNA-mediated *MYBL2* knockdown compared to a non-targeting siControl in A673, SK-N-MC, and RDES EwS cell lines, all genes were ranked by their mean log2 FC and a pre-ranked GSEA was performed with 1000 permutations^52^.

### GGAA-microsatellite analysis using HipSTR

EwS tumors and/or matched blood samples were collected with informed consent from EwS patients treated in the Hospital for Sick Children (SickKids) in Toronto, Canada, in accordance with Research Ethical Board (REB) guidelines (approval no. 1000053452). In addition, publicly available EwS reference samples from the International Cancer Genome Consortium (ICGC) with matched tumor/germline WGS data were used for analysis^6^. WGS was performed in all tumors and available matched germline samples using established protocols on Illumina instruments (paired-end 150/150 bp for the Toronto cohort, and paired-end 100/100 bp for the ICGC cohort). Paired-end FASTQ files were aligned to the human genome (hg19/GRCh37) using BWA-MEM (v.0.7.8). Indel realignment and base quality scores were recalibrated using the Genome Analysis Toolkit (v.2.8.1). For the Toronto cohort, published gene expression data were available from RNA-seq which was deposited at the European Genome-phenome Archive (EGA) under accession number EGAS00001003062; and for the ICGC cohort from matched Affymetrix HG-U133A or HG-U133Plus2.0 gene expression arrays (GSE37371; GSE7007; GSE34620). Affymetrix gene expression data were normalized separately for each chip type by RMA^53^ using custom brainarray CDF (v20, ENTREZ)^54^. Batch effects were removed using ComBat^50,51^. For eQTL analyses, only tumor samples with a minimum tumor purity of >60%, corresponding to TCGA standard tissue requirements (http://cancergenome.nih.gov/cancersselected/biospeccriteria), were used. Tumor purity estimates were made using the AscatNGS (Toronto cohort)^55^ or the ESTIMATE algorithm (ICGC cohort)^56^. To call the genotypes of the *MYBL2*-associated GGAA-microsatellite, we applied HipSTR (v.0.6.2)^23^ on the WGS data using a minimum threshold of ten reads. All genotypes passed the following HipSTR default filters: --min-call-qual 0.9; --max-call-flank-indel 0.15; --max-call-stutter 0.15; --min-call-allele-bias -2; --min-call-strand-bias -2.

### Human samples and ethics approval

Archived human tissue samples were retrieved from the Institute of Pathology of the LMU Munich (Germany) and the Gerhard-Domagk Institute of Pathology of the University Hospital of Münster (Germany). All patients provided written informed consent. Retrospective and blinded analysis of anonymized samples was carried out upon ethical approval of LMU Munich’s ethics committee (approval no. 550-16 UE).

### Tissue microarrays and immunohistochemistry

Formalin-fixed paraffin-embedded samples were collected at the Institute of Pathology of the LMU Munich^57^. We harvested at least two cores per sample with a core-diameter of 1 mm from all blocks to construct tissue microarrays. All EwS samples showed cytogenetic evidence for a translocation of the *EWSR1* gene either as determined by fluorescence in situ hybridization and/or qRT-PCR. The samples were reviewed by a reference pathologist. Four-micrometer sections were cut for immunohistochemistry and antigen retrieval was performed with microwave treatment using the antigen retrieval ProTaqs I Antigen-Enhancer (Quartett) for p-MYBL2 or the Target Retrieval Solution (Agilent Technologies) for cleaved caspase 3. In total, 7.5% aqueous H_2_O_2_ solution (room temperature) and blocking serum from the corresponding kits were used for 20 min for blockage of endogenous peroxidase. Then slides were incubated for 60 min with the primary antibodies anti-p-MYBL2 (1:100 dilution; Abcam, ab76009) and anti-cleaved caspase 3 (1:100 dilution, Cell Signaling, #9661). Afterwards slides were incubated with a secondary anti-rabbit IgG antibody (MP-7401, ImmPress Reagent Kit, Peroxidase-conjugated) followed by subsequent target detection using DAB+chromogen (Agilent Technologies). Slides were counterstained with hematoxylin Gill’s Formula (H-3401; Vector).

### Evaluation of immunoreactivity and quantification of mitoses

Evaluation of p-MYBL2 immunostaining was carried out semi-quantitatively by a blinded observer in analogy to the Immune Reactive Score (IRS), which is used routinely by pathologists for quantification of hormone receptor expression in mammary carcinoma, ranging from 0 to 12 as described^58^. The intensity of p-MYBL2 immunoreactivity (score 0 = none, score 1 = low, score 2 = intermediate, and score 3 = strong) and the percentage of cells stained with each intensity (score 0 = 0%, score 1 = 0–9%, score 2 = 10–50%, score 3 = 51–80%, and score 4 = 81–100%) was determined per high-power field (×40). The product of the predominant intensity score and its percentage score defined the final IRS. For cleaved caspase 3 immunostaining, automated quantification of the percentage of positive high-power field area was performed using Fiji (ImageJ)^40,41^. Mitoses were quantified in HE-stained slides by a blinded observer per high-power field. Final scores/quantifications were determined by examination of 4–16 high-power fields of at least one section for each sample.

### Statistical analysis and software

Statistical data analysis was performed using GraphPad PRISM 5 (GraphPad Software Inc., CA, USA) on the raw data. If not otherwise specified in the figure legends comparison of two groups in functional in vitro experiments was carried out using a two-tailed Mann–Whitney test; ***P < 0.001, **P < 0.01, *P < 0.05. Comparison of three groups with data in ordinal scale was performed using Kruskal–Wallis test. If not otherwise specified in the figure legends, data are presented as dot plots with horizontal bars representing means, and whiskers representing the standard error of the mean (SEM). Sample size for all in vitro experiments was chosen empirically. In Kaplan–Meier survival analyses, curves were calculated from all individual survival times of patients or mice, respectively. Curves were compared by Mantel–Haenszel test to detect significant differences between the groups. For in vivo experiments, sample size was predetermined using power calculations with *β* = 0.8 and *α* = 0.05 based on preliminary data and in compliance with the 3R system (replacement, reduction, refinement).

### Reporting summary

Further information on research design is available in the Nature Research Reporting Summary linked to this article.

## Supplementary information

Supplementary Information

Description of Additional Supplementary Files

Supplementary Data 1

Supplementary Data 2

Supplementary Data 3

Supplementary Data 4

Supplementary Data 5

Supplementary Data 6

Supplementary Data 7

Supplementary Data 8

Supplementary Data 9

Supplementary Data 10

Supplementary Data 11

Reporting Summary

Source Data

## Data Availability

RNA-seq and ChIP-seq data have been deposited at the Gene Expression Omnibus (GEO) under the accession code GSE119972. Microarray data of 166 primary EwS tumors are available from the GEO website under the accession codes GSE63157 (ref. ^59^), GSE34620 (ref. ^60^), GSE12102 (ref. ^61^), GSE17618 (ref. ^62^). Survival data were crossed with gene expression microarray data (Affymetrix HG-U133A2.0) generated in A673/TR/shEF1 cells (GSE27524 (ref. ^63^); 53 h DOX-treatment). Publicly available ENCODE SK-N-MC DNAse-seq data (GSM736570 (ref. ^21^)) and pre-processed A673 and SK-N-MC ChIP-seq data (GSE61944 (ref. ^22^) were retrieved from the GEO and displayed in the UCSC genome browser. The following samples were used: GSM1517544 SK-N-MC_shGFP_48h_FLI1; GSM1517553 SK-N-MC_shFLI1_48h_FLI1; GSM1517569 A673_shGFP_48h_FLI1; GSM1517572 A673_shFLI1_48h_FLI1; GSM1517548 SK-N-MC_shGFP_96h_H3K4me1; GSM1517557 SK-N-MC_shFLI1_96h_H3K4me1; GSM1517545 SK-N-MC_shGFP_48h_H3K27ac; GSM1517554 SK-N-MC_shFLI1_48h_H3K27ac; GSM1517568 A673 whole-cell extract (WCE). For gene expression analysis of tumors for which matched germline/tumor WGS was available, published gene expression data from the Toronto cohort was available from RNA-seq which was deposited at the European Genome-phenome Archive (EGA) under accession number EGAS00001003062(ref. ^12^); and for the ICGC cohort from matched Affymetrix HG-U133A or HG-U133Plus2.0 gene expression arrays (GSE37371; GSE7007 (ref. ^64^); GSE34620 (ref. ^60^)). The source data underlying Figs. 1a–c, 1e–f, 2a–g, 2i, 3a–b, 3d–g, 4a–c, and Supplementary Figs. 1a–b, 1d–j, 2a–d, 2g, 3a, 3c–d, 4a–g, and 5a–e are provided as a Source Data file. All the other data supporting the findings of this study are available within the article and its supplementary information files and from the corresponding author upon reasonable request. A reporting summary for this article is available as a Supplementary Information file.

## Code availability

Custom code is available from the corresponding author upon reasonable request.
