# Supplementary material for: Cooperation of cancer drivers with regulatory germline variants shapes clinical outcomes
Source: Nat Commun. 2019 Sep 11;10:4128. doi: 10.1038/s41467-019-12071-2 (PMC6739408; doi:10.1038/s41467-019-12071-2)
Supplement: Supplementary file 1 — Supplementary Information [file 41467_2019_12071_MOESM1_ESM.pdf]

Supplementary Information

Cooperation of cancer drivers with regulatory germline variants shapes clinical outcomes

Musa *et al.*

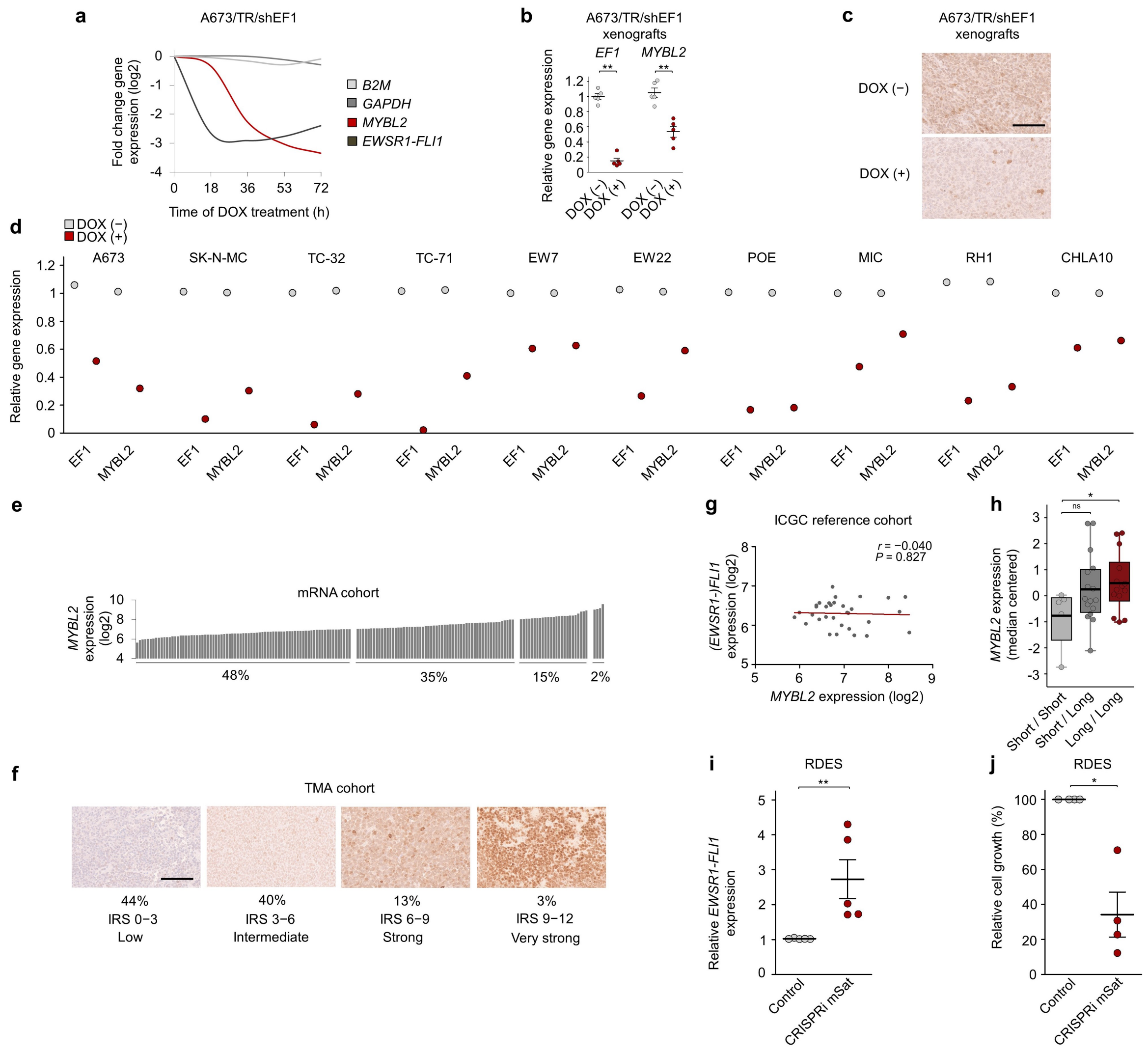

Supplementary Figure 1 | MYBL2 is regulated by EWSR1-FLI1 via a polymorphic GGAA-microsatellite a, Expression fold change of indicated genes in A673/TR/shEF1 cells containing a DOX-inducible shRNA construct against *EWSR1-FLI1*, determined by Affymetrix microarrays at indicated time points after start of DOX-addition, $n \geq 2$ technical replicates per condition. **b**, *Ex vivo* analysis of *EWSR1-FLI1* (*EF1*) and *MYBL2* expression by qRT-PCR in A673/TR/shEF1 xenografts with/without DOX-treatment. Horizontal bars represent means, and whiskers the SEM, n=5 samples per condition. **c,** Representative IHC micrographs of xenografts stained for p-MYBL2 described in (b). Scale bar is 100 µm. **d,** *EWSR1-FLI1* (*EF1*) and *MYBL2* expression measured by qRT-PCR in ten EwS cell lines containing a DOX-inducible shRNA against *EWSR1-FLI1*. Dots represent relative expression levels, n=1 biologically independent experiment. **e,f** Ranked log2-transformed *MYBL2* expression intensities in 166 primary EwS tumors as determined by Affymetrix microarrays (e) and representative IHC micrographs of TMAs stained for p-MYBL2 comprising 208 primary EwS (f). Scale bar is 100 µm. Percentages of tumors showing either low, intermediate, strong, or

very strong *MYBL2* expression are reported. **g,** Linear regression of *(EWSR1-)FLI1* expression onto *MYBL2* expression in the International Cancer Genome Consortium (ICGC) reference expression dataset[3] (Affymetrix HG-U133Plus2 microarrays) comprising 32 primary EwS tumors with *EWSR1-FLI1* translocation. *FLI1* expression served as a surrogate for *EWSR1-FLI1*, since wildtype *FLI1* is virtually not expressed in EwS. **h,** *MYBL2* expression in 35 primary EwS depending on the repeat length of the *MYBL2*-associated GGAA-microsatellite. Haplotypes with ≤13 consecutive GGAA-repeats were considered as 'short', and haplotypes with >13 consecutive GGAA-repeats as 'long'. Horizontal bars represent means, the upper and lower hinges the 75th and 25th percentile (interquartile range), respectively, and whiskers minimum and maximum expression values, $n \geq 5$ samples per group. **i,** Analysis of relative *EWSR1-FLI1* expression in RDES cells after CRISPRi-mediated targeting of the *MYBL2*-associated GGAA-microsatellite. Horizontal bars represent means, and whiskers the SEM, $n=5$ biologically independent experiments. **j,** Relative growth of RDES cells after CRISPRi-mediated targeting of the *MYBL2*-associated GGAA-microsatellite. Horizontal bars represent means, and whiskers the SEM, $n=4$ biologically independent experiments.

Not significant, ns; ***$P<0.001$, **$P<0.01$, *$P<0.05$; P values determined via two-tailed Mann-Whitney test. Source data are provided as a Source Data file.

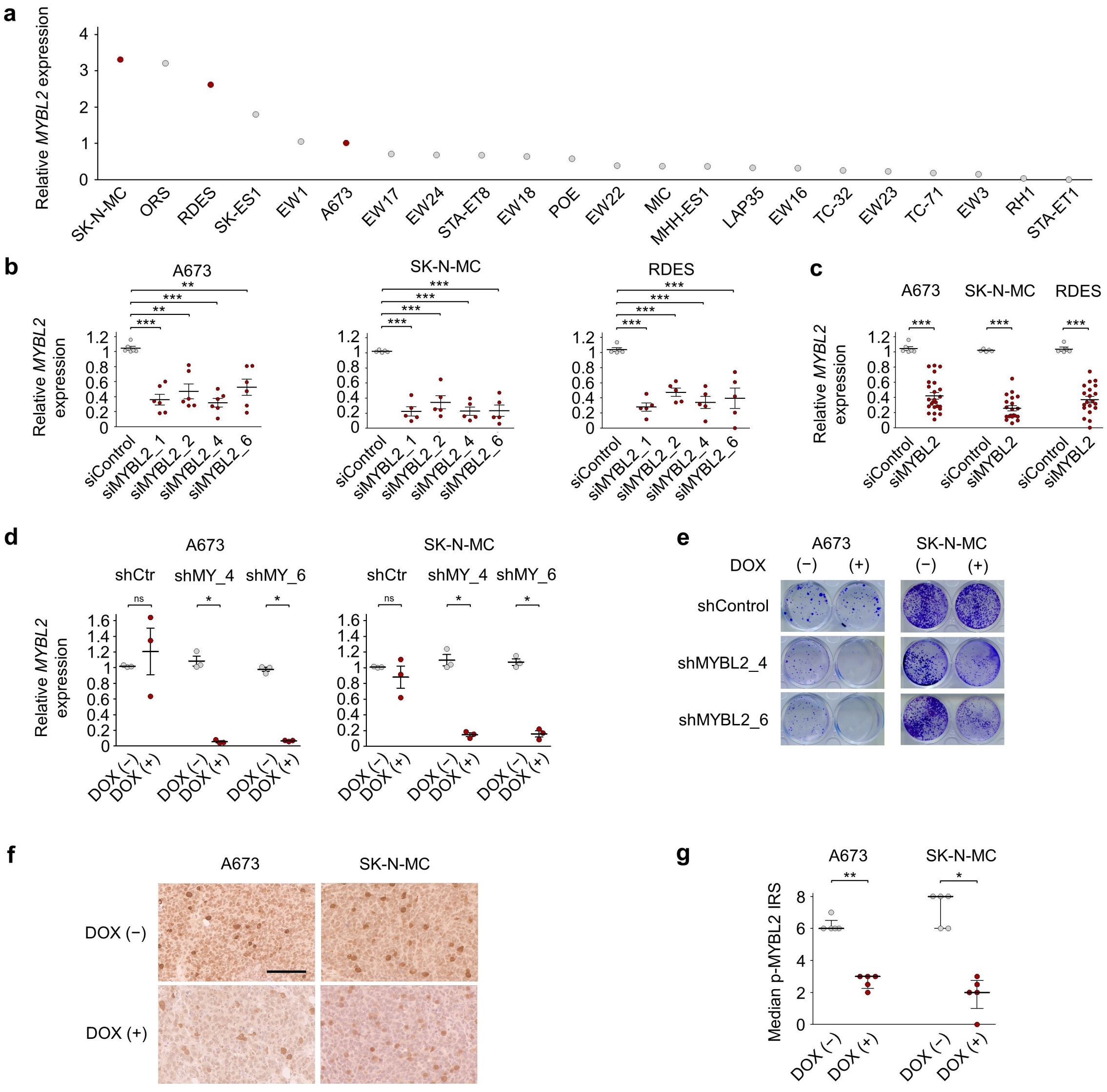

Supplementary Figure 2 | Silencing of MYBL2 inhibits clonogenic growth of EwS cells

a, Relative *MYBL2* expression levels (represented as dots) in 22 EwS cell lines normalized to A673 as measured by qRT-PCR, *n*=1 biologically independent experiment. Red color indicates the cell lines that were used for the majority of the reported experiments. **b,** Relative *MYBL2* expression as measured by qRT-PCR after transfection of A673, SK-N-MC and RDES EwS cell lines with either four different specific siRNAs directed against *MYBL2* or a non-targeting siControl. Horizontal bars represent means, and whiskers the SEM, *n*≥5 biologically independent experiments. **c,** Summary of four different specific siRNAs directed against *MYBL2* shown in (b). Horizontal bars represent means, and whiskers the SEM, *n*≥5 biologically independent experiments. **d,** Relative *MYBL2* expression as measured by qRT-PCR of A673 and SK-N-MC cells containing either DOX-inducible specific shRNA constructs directed against *MYBL2* or a non-targeting shControl, grown with/without DOX. Horizontal bars represent means, and whiskers the SEM, *n*=3 biologically independent experiments; *P* values

determined via one-tailed Mann-Whitney test. **e,** Representative colony forming assays of A673 and SK-N-MC cells containing either DOX-inducible specific shRNA constructs directed against MYBL2 (shMY_4 refers to shMYBL2_4 and shMY_6 refers to shMYBL2_6) or a non-targeting shControl (shCtr). Cells were grown either with or without DOX. **f,** Representative IHC micrographs of xenografts of A673 and SK-N-MC cells containing a DOX-inducible specific shRNA construct directed against *MYBL2* stained for p-MYBL2. Mice were treated with/without addition of DOX to the drinking water. Scale bar is 100 µm. **g,** Quantification of p-MYBL2 staining as described in (f). Horizontal bars represent medians, and whiskers represent the interquartile range, n=5 samples per condition.

Not significant, ns; ***P<0.001, **P<0.01, *P<0.05; P values determined via two-tailed Mann-Whitney test. Source data are provided as a Source Data file.

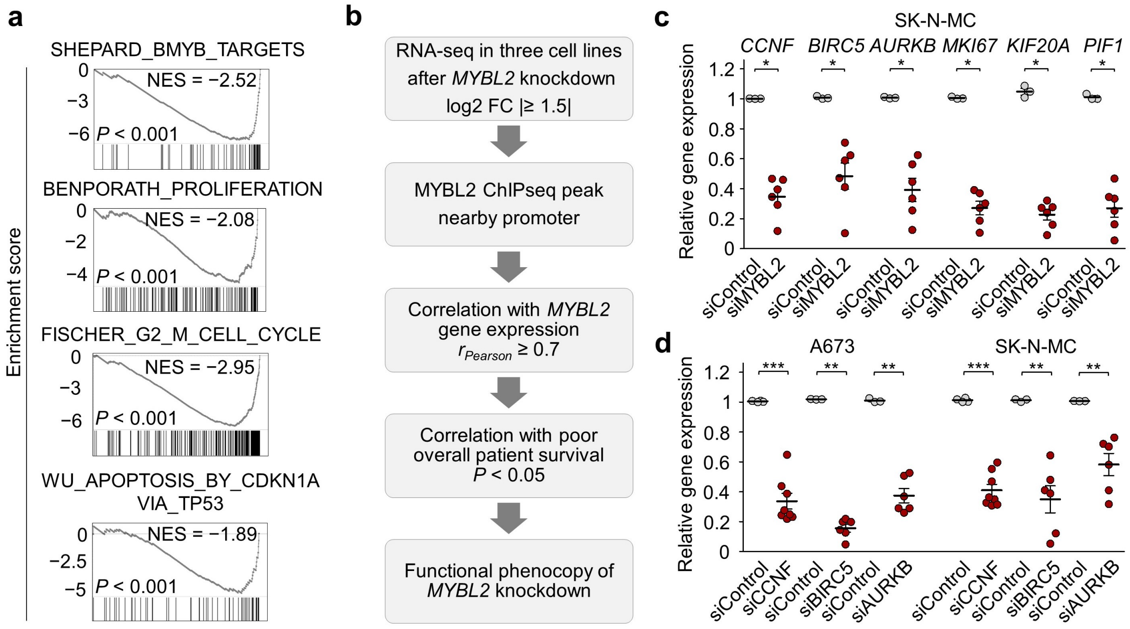

Supplementary Figure 3 | MYBL2 confers a pro-proliferative gene expression signature to EwS cells

a, Selected negatively enriched gene-sets in RNA-seq data after siRNA-mediated *MYBL2* knockdown compared to a non-targeting siControl (summary of three cell lines, *n*=3 technical replicates per cell line). **b,** Flow-chart showing the algorithm used to identify the top functionally relevant downstream target genes of MYBL2. **c,** Validation of downregulation of selected DEGs by qRT-PCR after *MYBL2* knockdown in SK-N-MC EwS cells. Horizontal bars represent means, and whiskers the SEM, *n*=3 biologically independent experiments. **d,** Relative gene expression of *CCNF*, *BIRC5* and *AURKB* expression as measured by qRT-PCR after transfection of A673 and SK-N-MC EwS cell lines with either two different specific siRNAs directed against *CCNF*, *BIRC5* or *AURKB* (summary of two siRNAs is shown) or a non-targeting siControl. Horizontal bars represent means, and whiskers the SEM, *n*≥3 biologically independent experiments.

****P*<0.001, ***P*<0.01, **P*<0.05; *P* values determined via two-tailed Mann-Whitney test. Source data are provided as a Source Data file.

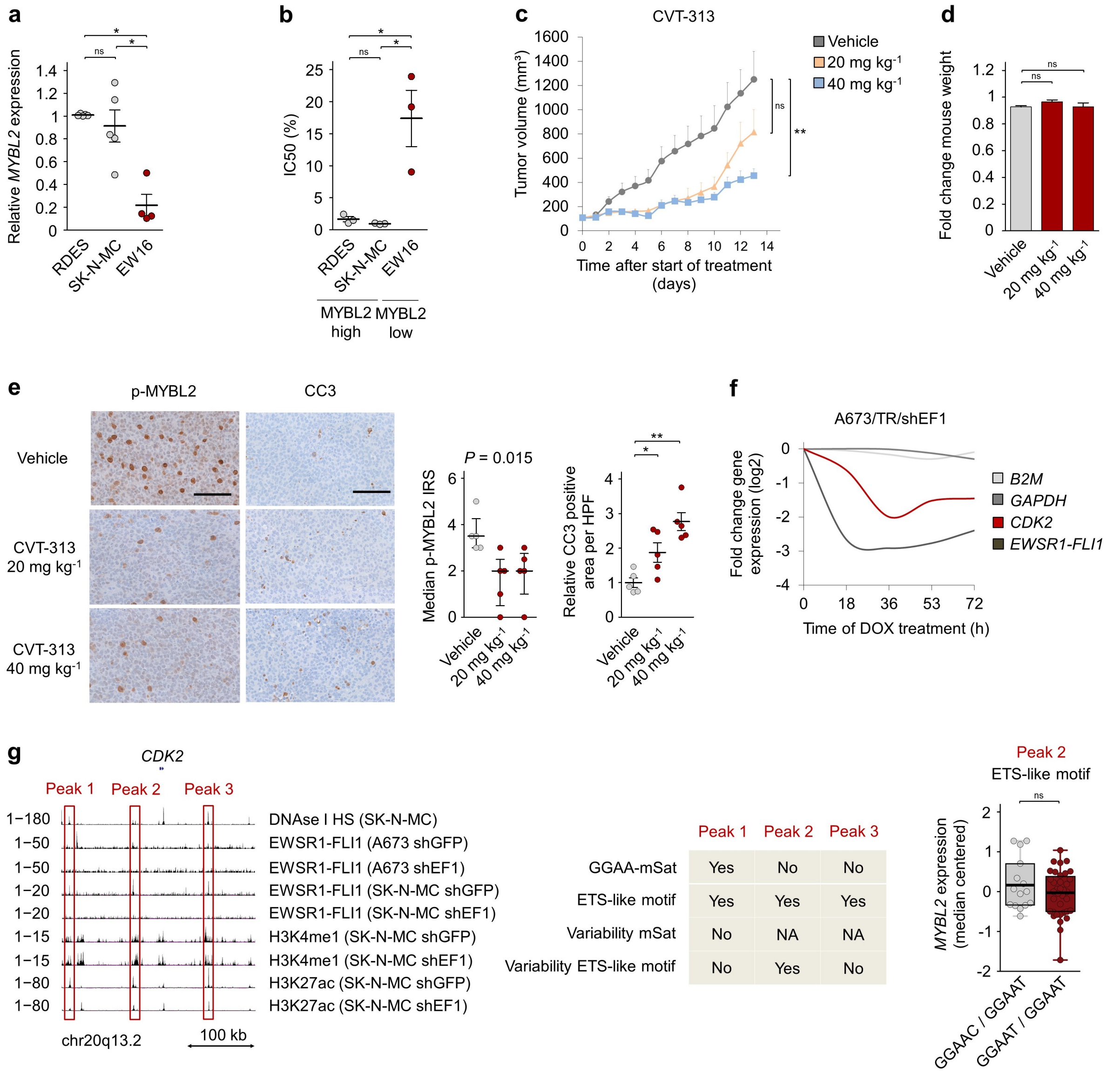

Supplementary Figure 4 | MYBL2 sensitizes EwS cells for CDK2 inhibition

a, *MYBL2* expression analysis in indicated EwS cells by qRT-PCR. Horizontal bars represent means, and whiskers the SEM, $n \geq 4$ biologically independent experiments. **b,** Analysis of IC50 of the CDK2 inhibitor NU6140 in RDES and SK-N-MC (both *MYBL2* high) and EW16 (*MYBL2* low) cells. Horizontal bars represent means, and whiskers the SEM, $n=3$ biologically independent experiments. **c,** Volume curves of A673 xenografts containing a DOX-inducible shMYBL2 construct (without DOX treatment), treated with either vehicle or CVT-313 (20 or 40 mg kg^{-1}). Mice were randomized to treatment groups when tumors were palpable. Mean tumor volume per condition is shown, whiskers represent the SEM, $n=5$ animals per condition; *P* values determined via two-tailed Mann-Whitney test. **d,** Fold change (FC) of mouse weight before/after treatment with CDK2 inhibitors (up to 40 mg kg^{-1} of CVT-313 or NU6140) for 14 days, $n \geq 13$ animals per condition. **e,** Left: Representative IHC micrographs of p-MYBL2 and

cleaved caspase 3 (CC3) staining of A673/TR/shMYBL2 xenografts (without DOX treatment) treated with either vehicle or CVT-313. Scale bar is 100 µm. Right: Quantification of positivity for p-MYBL2 and CC3, respectively. Horizontal bars represent medians or means, and whiskers interquartile ranges or SEM for p-MYBL2 or CC3, respectively, n=5 samples per condition; P values determined via Kruskal-Wallis test (p-MYBL2) or two-tailed Mann-Whitney test (CC3). **f,** Expression of indicated genes in A673/TR/shEF1 cells containing a DOX-inducible shRNA against *EWSR1-FLI1*, determined by Affymetrix microarrays at different time points after start of DOX-addition to the media, n≥2 technical replicates per condition. **g,** Epigenetic profile of the *CDK2* locus in indicated EwS cells transduced with either a control shRNA (shGFP) or a specific shRNA against *EWSR1-FLI1* (shEF1). eQTL analysis was performed for the given peaks if genetic variability was detected in WGS data of EwS tumors. Horizontal bars represent means, the upper and lower hinges the 75th and 25th percentile (interquartile range), respectively, and whiskers minimum and maximum expression values; n≥15 samples per group.

Not significant, ns; *P<0.05; **P<0.01; P values determined via two-tailed Mann-Whitney test. Source data are provided as a Source Data file.

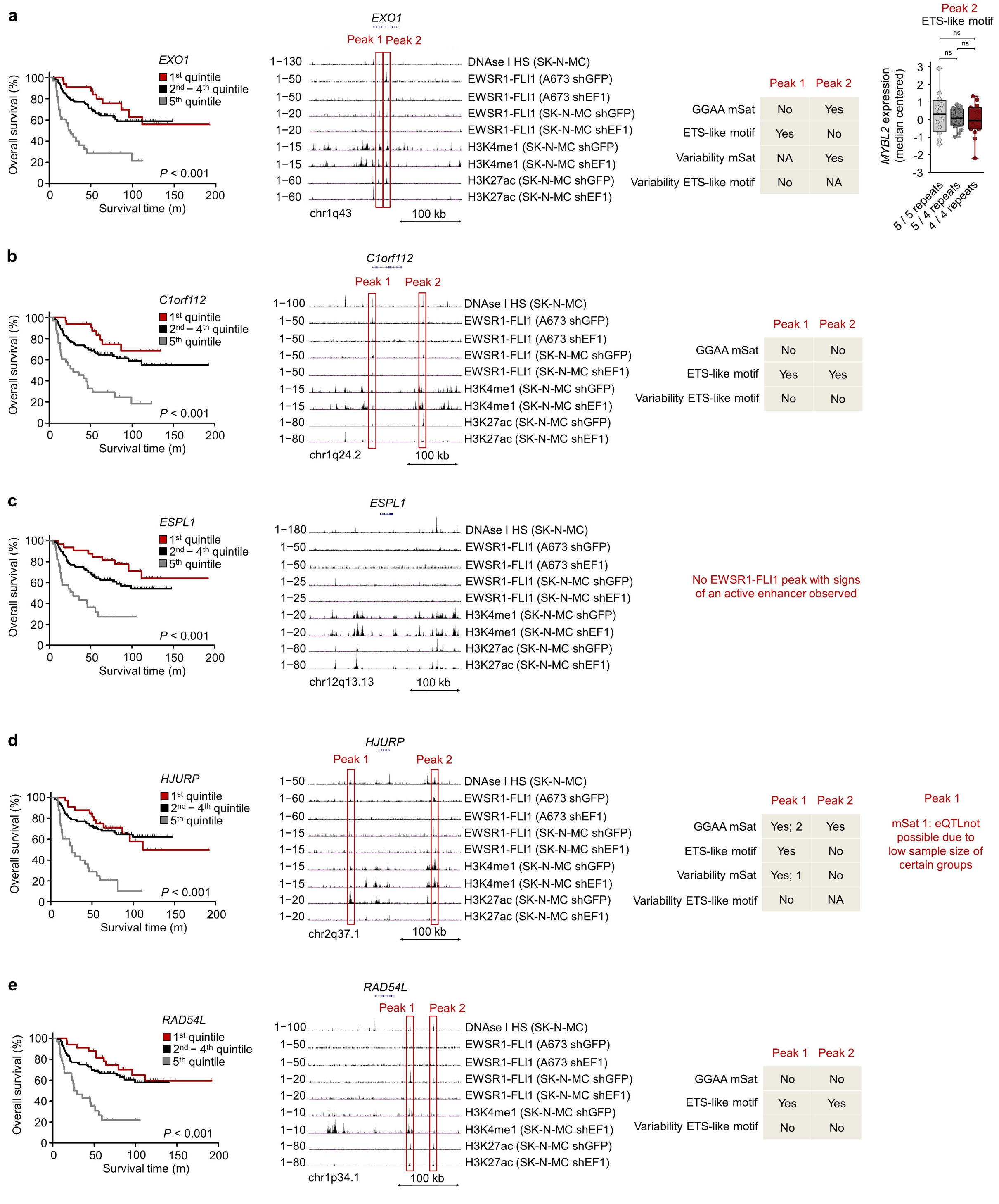

Supplementary Figure 5 | Analysis of the epigenetic profiles and potential eQTL effects of additional EWSR1-FLI1-regulated genes associated with clinical outcomes

Kaplan-Meier survival analysis of 166 EwS patients, as well as epigenetic profile of indicated EwS cells transfected with either a control shRNA (shGFP) or a specific shRNA against *EWSR1-FLI1* (shEF1) from published DNAse-seq (DNAse I hypersensitivity (HS)) data and ChIP-seq data for EWSR1-FLI1, H3K4me1 and H3K27ac for loci of **a,** *EXO1*, **b,** *C1orf112*, **c,** *ESPL1*, **d,** *HJURP*, **e,** *RAD54L*. eQTL analysis was performed for the given loci if genetic

variability was detected in WGS data from EwS tumor samples. Horizontal bars represent means, the upper and lower hinges the 75th and 25th percentile (interquartile range), respectively, and whiskers minimum and maximum expression values, $n \geq 12$ samples per group.
Not significant, ns; *P* values of survival analyses were determined via Mantel-Haenszel test. Other *P* values determined via two-tailed Mann-Whitney test. Source data are provided as a Source Data file.

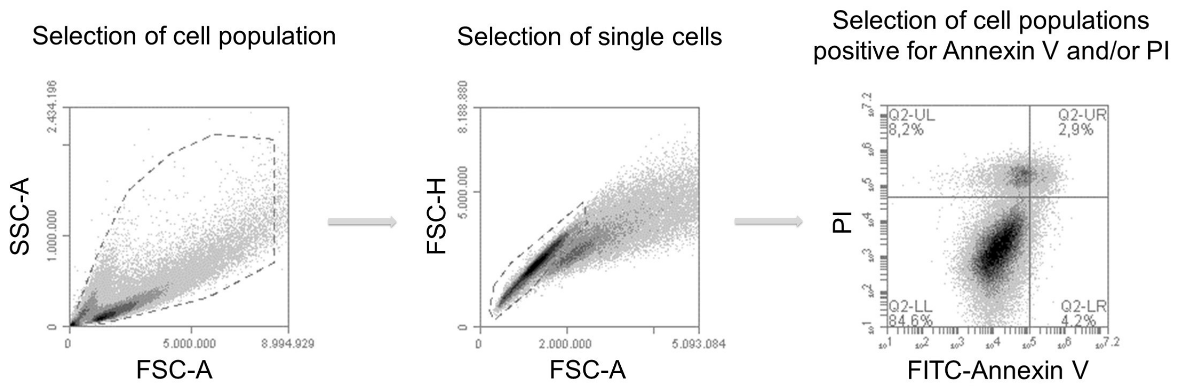

Supplementary Figure 6 | Example of the gating strategy for flow cytometric analysis of Annexin V/PI-positivity in EwS cells.
