# Supplementary material for: Cooperation of cancer drivers with regulatory germline variants shapes clinical outcomes
Source: Nat Commun. 2019 Sep 11;10:4128. doi: 10.1038/s41467-019-12071-2 (PMC6739408; doi:10.1038/s41467-019-12071-2)
Supplement: Supplementary file 2 — Description of Additional Supplementary Files [file 41467_2019_12071_MOESM2_ESM.pdf]

Description of Additional Supplementary Files

File Name: Supplementary Data 1
Description: Log2 gene expression fold change in a microarray dataset of A673 Ewing sarcoma cells harboring a doxycycline-inducible shRNA against EWSR1-FLI1 (A673/TR/shEF1) profiled with/without addition of doxycycline for 53h.

File Name: Supplementary Data 2
Description: Significance levels of the listed genes for association with overall patient survival stratifying patients by expression quintiles in a microarray dataset of 166 primary Ewing sarcomas with matched clinical annotation.

File Name: Supplementary Data 3
Description: Top 10 additional EWSR1-FLI1 regulated genes with significant association with overall patient survival.

File Name: Supplementary Data 4
Description: Number of consecutive GGAA-repeats of the MYBL2 associated GGAA-microsatellite in whole-genome sequencing data for matched tumor and germline DNA.

File Name: Supplementary Data 5
Description: Gene-set enrichment analysis of MYBL2 co-expressed genes in 166 primary Ewing sarcomas.

File Name: Supplementary Data 6
Description: Gene-set enrichment analysis of differentially expressed genes upon siRNA-mediated knockdown of MYBL2 in three different Ewing sarcoma cell lines compared to treatment with a non-targeting siControl.

File Name: Supplementary Data 7
Description: Most significantly differentially expressed genes comparing siRNAmediated knockdown of MYBL2 to treatment with a non-targeting siControl in three Ewing sarcoma cell lines.

File Name: Supplementary Data 8
Description: Called peaks of MYBL2 ChIP-seq in A673 Ewing sarcoma cells.

File Name: Supplementary Data 9

Description: Correlation of MYBL2 expression with the expression of genes given in Supplementary Data 6 that exhibit MYBL2 promoter binding.

File Name: Supplementary Data 10
Description: Significance levels of the listed genes for association with overall patient survival stratifying patients by median expression in a microarray dataset of 166 primary Ewing sarcomas with matched clinical annotation.

File Name: Supplementary Data 11
Description: Oligonucleotide sequences.
