# Supplementary material for: Cooperation of cancer drivers with regulatory germline variants shapes clinical outcomes
Source: Nat Commun. 2019 Sep 11;10:4128. doi: 10.1038/s41467-019-12071-2 (PMC6739408; doi:10.1038/s41467-019-12071-2)
Supplement: Supplementary file 14 — Reporting Summary [file 41467_2019_12071_MOESM14_ESM.pdf]

nature research

Corresponding author(s): Thomas G.P. Grünewald

Last updated by author(s): Jul 29, 2019

Reporting Summary

Nature Research wishes to improve the reproducibility of the work that we publish. This form provides structure for consistency and transparency in reporting. For further information on Nature Research policies, see Authors & Referees and the Editorial Policy Checklist.

Statistics

For all statistical analyses, confirm that the following items are present in the figure legend, table legend, main text, or Methods section.

n/a	Confirmed	
☐	☒	The exact sample size (*n*) for each experimental group/condition, given as a discrete number and unit of measurement
☐	☒	A statement on whether measurements were taken from distinct samples or whether the same sample was measured repeatedly
☐	☒	The statistical test(s) used AND whether they are one- or two-sided<br>*Only common tests should be described solely by name; describe more complex techniques in the Methods section.*
☒	☐	A description of all covariates tested
☐	☒	A description of any assumptions or corrections, such as tests of normality and adjustment for multiple comparisons
☐	☒	A full description of the statistical parameters including central tendency (e.g. means) or other basic estimates (e.g. regression coefficient) AND variation (e.g. standard deviation) or associated estimates of uncertainty (e.g. confidence intervals)
☐	☒	For null hypothesis testing, the test statistic (e.g. *F*, *t*, *r*) with confidence intervals, effect sizes, degrees of freedom and *P* value noted<br>*Give P values as exact values whenever suitable.*
☒	☐	For Bayesian analysis, information on the choice of priors and Markov chain Monte Carlo settings
☒	☐	For hierarchical and complex designs, identification of the appropriate level for tests and full reporting of outcomes
☐	☒	Estimates of effect sizes (e.g. Cohen's *d*, Pearson's *r*), indicating how they were calculated

Our web collection on statistics for biologists contains articles on many of the points above.

Software and code

Policy information about availability of computer code

Data collection	GraphPad PRISM 5 and 7, HipSTR, Micorsoft Excel v16.16.5, GenEx, Integrative Genomics Viewer (IGV), UCSC Genome Browser, GSEA application (Broad Institute), Fiji (Image J), Accuri C6 CFlow Plus, PAVIS. For further details see Methods section.
Data analysis	GraphPad PRISM 5 and 7, HipSTR, Micorsoft Excel v16.16.5, GenEx, Integrative Genomics Viewer (IGV), UCSC Genome Browser, GSEA application (Broad Institute), Accuri C6 CFlow Plus, PAVIS. For further details see Methods section.

For manuscripts utilizing custom algorithms or software that are central to the research but not yet described in published literature, software must be made available to editors/reviewers. We strongly encourage code deposition in a community repository (e.g. GitHub). See the Nature Research guidelines for submitting code & software for further information.

Data

Policy information about availability of data

All manuscripts must include a data availability statement. This statement should provide the following information, where applicable:

- Accession codes, unique identifiers, or web links for publicly available datasets
- A list of figures that have associated raw data
- A description of any restrictions on data availability

RNA-seq and ChIP seq data have been deposited at the Gene Expression Omnibus (GEO) under the accession code GSE119972 [hyperlink not yet available].
Microarray data of 166 primary EwS tumors are available from the GEO website under the accession codes GSE6315759[https://www.ncbi.nlm.nih.gov/geo/query/acc.cgi?acc=gse63157], GSE3462060[https://www.ncbi.nlm.nih.gov/geo/query/acc.cgi?acc=gse34620], GSE1210261[https://www.ncbi.nlm.nih.gov/geo/query/acc.cgi?acc=GSE12102], GSE1761862[https://www.ncbi.nlm.nih.gov/geo/query/acc.cgi?acc=GSE17618].
Survival data were crossed with gene expression microarray data (Affymetrix HG-U133A2.0) generated in A673/TR/shEF1 cells (GSE2752463[https://www.ncbi.nlm.nih.gov/geo/query/acc.cgi?acc=GSE27524]; 53h DOX-treatment).
Publicly available ENCODE SK-N-MC DNAse-seq data (GSM73657021[https://www.ncbi.nlm.nih.gov/geo/query/acc.cgi?acc=GSM736570]) and pre-processed A673 and SK-N-MC ChIP-seq data (GSE6194422[https://www.ncbi.nlm.nih.gov/geo/query/acc.cgi?acc=GSE61944]) were retrieved from the GEO and displayed in the UCSC genome browser. The following samples were used: GSM1517544 SK-N-MC_shGFP_48h_FLI1; GSM1517553 SK-N-MC_shFLI1_48h_FLI1; GSM1517569

A673_shGFP_48h_FLI1; GSM1517572 A673_shFLI1_48h_FLI1; GSM1517548 SK-N-MC_shGFP_96h_H3K4me1; GSM1517557 SK-N-MC_shFLI1_96h_H3K4me1; GSM1517545 SK-N-MC_shGFP_48h_H3K27ac; GSM1517554 SK-N-MC_shFLI1_48h_H3K27ac; GSM1517568 A673 whole cell extract (WCE).
For gene expression analysis of tumors for which matched germline/tumor WGS was available, published gene expression data from the Toronto cohort was available from RNA-seq which was deposited at the European Genome-phenome Archive (EGA) under accession number EGAS0000100306212 [https://www.ebi.ac.uk/ega/studies/EGAS00001003062]; and for the ICGC cohort from matched Affymetrix HG-U133A or HG-U133Plus2.0 gene expression arrays (GSE37371[https://www.ncbi.nlm.nih.gov/geo/query/acc.cgi?acc=GSE37371]; GSE700764[https://www.ncbi.nlm.nih.gov/geo/query/acc.cgi?acc=GSE7007]; GSE3462060[https://www.ncbi.nlm.nih.gov/geo/query/acc.cgi?acc=gse34620]).
The source data underlying Figs 1a–c, 1e–f, 2a–g, 2i, 3a–b, 3d–g, 4a–c, and Supplementary Figs 1a–b, 1d–j, 2a–d, 2g, 3a, 3c–d, 4a–g, and 5a–e are provided as a Source Data file.
All the other data supporting the findings of this study are available within the article and its supplementary information files and from the corresponding author upon reasonable request. A reporting summary for this article is available as a Supplementary Information file.

Field-specific reporting

Please select the one below that is the best fit for your research. If you are not sure, read the appropriate sections before making your selection.

☒ Life sciences ☐ Behavioural & social sciences ☐ Ecological, evolutionary & environmental sciences

For a reference copy of the document with all sections, see nature.com/documents/nr-reporting-summary-flat.pdf

Life sciences study design

All studies must disclose on these points even when the disclosure is negative.

Sample size	Sample size for all in vitro experiments was chosen empirically. For in vivo experiments, sample size was predetermined using power calculations with ß=0.8 and P<0.05 based on preliminary data and in compliance with the 3R system (replacement, reduction, refinement).
Data exclusions	No data were excluded from the analyses.
Replication	All attempts at replication yielded similar results.
Randomization	Where applicable (in vivo experiments), animals were allocated to groups in an unbiased manner.
Blinding	Analysis of human tissue samples was carried out blinded. For animal treatment studies, drug adminsitration was only possible in an unblinded manner.

Reporting for specific materials, systems and methods

We require information from authors about some types of materials, experimental systems and methods used in many studies. Here, indicate whether each material, system or method listed is relevant to your study. If you are not sure if a list item applies to your research, read the appropriate section before selecting a response.

Materials & experimental systems

n/a	Involved in the study	
☐	☒	Antibodies
☐	☒	Eukaryotic cell lines
☒	☐	Palaeontology
☐	☒	Animals and other organisms
☐	☒	Human research participants
☐	☒	Clinical data

Methods

n/a	Involved in the study	
☐	☒	ChIP-seq
☐	☒	Flow cytometry
☒	☐	MRI-based neuroimaging

Antibodies

Antibodies used	For immunohistochemistry analyses, the following primary antibodies were used:<br>anti-p-MYBL2 (Abcam, ab76009); cleaved caspase 3 (Cell signaling, #9661)<br><br>For Western blot analyses, the following primary antibodies were used:<br>Rabbit monoclonal anti-FLI1 antibody (ab133485, Abcam), rabbit polyclonal anti-MYBL2 antibody (sc-725, Santa Cruz) and mouse monoclonal anti-ß-actin (A-5316, Sigma-Aldrich).<br><br>For ChIP-Seq analyses, the following antibodies were used:<br>Monoclonal ChIP-grade rabbit anti-MYBL2 antibody (Abcam, ab76009, lot GR113270-6); polyclonal ChIP-grade rabbit anti-FLI1 antibody (Abcam, ab15289, lot GR293950-1); rabbit IgG (Diagenode, C15410206, RIG001).

Validation

Sepcificity of the used antibodies against human antigens was tested by knockdown experiments of the corresponding antigen and subsequent Western blot and/or immunohistochemistry analyses. In addition, antibodies were validated for the given application by the manufacturer as stated in the respective datasheets and cited references.

Eukaryotic cell lines

Policy information about cell lines

Cell line source(s)

A673 and HEK293T cells were purchased from American Type Culture Collection (ATCC). MHH-ES1, RDES, RH1, SK-ES1 and SK-N-MC cells were provided by the German Collection of Microorganisms and Cell lines (DSMZ). TC 32, TC-71, CHLA-10 cells were kindly provided by the Children's Oncology Group (COG) and EW1, EW3, EW7, EW16, EW17, EW18, EW22, EW23, EW24, LAP35, MIC, ORS, POE, STA-ET1, STA-ET8 cells were provided by O. Delattre (Institute Curie, Paris). A673/TR/shEF1 cells were kindly provided by J. Alonso (Madrid, Spain).

Authentication

Cell lines were authenticated by STR-profiling and if applicable by detection of specific fusion oncogenes by qRT-PCR.

Mycoplasma contamination

Cells were tested for mycoplasma contamination by nested PCR.

Commonly misidentified lines
(See ICLAC register)

SK-N-MC

Animals and other organisms

Policy information about studies involving animals; ARRIVE guidelines recommended for reporting animal research

Laboratory animals

NSG mice were obtained from Jackson Laboratory via Charles River Laboratories and bred under a MTA with LMU Munich.

Wild animals

No wild animals were used in this study.

Field-collected samples

Not applicable.

Ethics oversight

Experiments were approved by the government of Upper Bavaria and conducted in accordance with ARRIVE guidelines, recommendations of the European Community (86/609/EEC), and UKCCCR (guidelines for the welfare and use of animals in cancer research).

Note that full information on the approval of the study protocol must also be provided in the manuscript.

Human research participants

Policy information about studies involving human research participants

Population characteristics

For survival analyses, public microarray data of 166 primary EwS tumors (accession codes: GSE63157, GSE34620, GSE12102, GSE17618) for which clinical annotations were available were downloaded from the GEO. Further information is given in the corresponding cited references.
Ewing sarcoma tumors and/or matched blood samples were collected with informed consent from Ewing sarcoma patients treated in the Hospital for Sick Children (SickKids) in Toronto, Canada, in accordance with Research Ethical Board (REB) guidelines (approval no. 1000053452). In addition, genomic data from a published reference cohort from the International Cancer Genome Consortium (ICGC) was analyzed with approval of the corresponding data access committee. Overall, the patients' clincial features and demographics were typical of Ewing sarcoma. The mean age at diagnosis was 15.8 years, the ratio of males/females was 1.2. Further details on the patients' characteristics are given in Supplementary Table 4.

Recruitment

Patients were recruited from the Hospital for Sick Children (SickKids) solid tumor bank on the basis of a confirmed diagnosis of Ewing sarcoma and the availability of both frozen tumor tissue as well as DNA from non neoplastic material (usually blood).

Ethics oversight

This study, involving profiling by genomic technologies, was approved by the Hospital for Sick Children Research Ethics Board (REB); approval no. 1000053542 .

Note that full information on the approval of the study protocol must also be provided in the manuscript.

Clinical data

Policy information about clinical studies
All manuscripts should comply with the ICMJE guidelines for publication of clinical research and a completed CONSORT checklist must be included with all submissions.

Clinical trial registration

The current study is neither a clinical study nor clinical trial.

Study protocol

Not applicable.

Data collection

Not applicable.

Outcomes

Not applicable.

ChIP-seq

Data deposition

☒ Confirm that both raw and final processed data have been deposited in a public database such as GEO.

☒ Confirm that you have deposited or provided access to graph files (e.g. BED files) for the called peaks.

Data access links<br>*May remain private before publication.*	GSE119972
Files in database submission	GSM3389599 A673_WT_MYBL2_A763C13<br>GSM3389600 A673_WT_input_A350C18
Genome browser session<br>(e.g. UCSC)	http://genome.ucsc.edu/cgi-bin/hgTracks?<br>hgS_doOtherUser=submit&hgS_otherUserName=slalami&hgS_otherUserSessionName=hg19_A673_MYBL2

Methodology

Replicates	No replicates were performed.
Sequencing depth	99,306,793 total reads, 56,834,390 uniquely mapped reads, single reads (101 bp)
Antibodies	Abcam rabbit anti-MYBL2 (ab76009, lot GR113270-6); Diagenode rabbit IgG (C15410206, lot RIG001)
Peak calling parameters	macs2 callpeak -B –t [BAM_FILE] -c [CTL_BAMFILE] -g hs -n A673_MYBL2 -qvalue 0.05
Data quality	66,965 peaks – FRIP (Fraction of Reads In Peaks) : 9 %
Software	samtools view –q 20 [BAM_FILE]; macs2 callpeak -B –t [BAM_FILE] -c [CTL_BAMFILE] -g hs -n A673_MYBL2 -qvalue 0.05<br>PAVIS

Flow Cytometry

Plots

Confirm that:

☒ The axis labels state the marker and fluorochrome used (e.g. CD4-FITC).

☒ The axis scales are clearly visible. Include numbers along axes only for bottom left plot of group (a 'group' is an analysis of identical markers).

☒ All plots are contour plots with outliers or pseudocolor plots.

☒ A numerical value for number of cells or percentage (with statistics) is provided.

Methodology

Sample preparation	Human Ewing sarcoma cells were trypsinized from cell culture dishes, washed with PBS, and stained for Annexin V-FITC and propidium iodide (PI) (analysis of apoptosis) or fixed with ethanol and stained with propidium iodide (PI) with addition of RNAse A (analysis of cell cycle).
Instrument	Accuri C6 flow cytometer.
Software	Accuri C6 CFlow Plus.
Cell population abundance	All flow cytometry experiments were done in pure human Ewing sarcoma cell lines without admixture of any other cell type.
Gating strategy	Gating included forward and side scatter plots for selection of the cell population (i.e. exclusion of debris), and subsequent selection of single cells. An example for the gating strategy is given in Supplementary Figure 6.

☒ Tick this box to confirm that a figure exemplifying the gating strategy is provided in the Supplementary Information.
